# Molecular networks involved in mouse cerebral corticogenesis and spatio-temporal regulation of *Sox4 *and *Sox11 *novel antisense transcripts revealed by transcriptome profiling

**DOI:** 10.1186/gb-2009-10-10-r104

**Published:** 2009-10-02

**Authors:** King-Hwa Ling, Chelsee A Hewitt, Tim Beissbarth, Lavinia Hyde, Kakoli Banerjee, Pike-See Cheah, Ping Z Cannon, Christopher N Hahn, Paul Q Thomas, Gordon K Smyth, Seong-Seng Tan, Tim Thomas, Hamish S Scott

**Affiliations:** 1 Molecular Medicine Division, The Walter and Eliza Hall Institute of Medical Research, Royal Parade, Parkville, Victoria 3052, Australia; 2 The School of Medicine, The University of Adelaide, SA, 5005, Australia; 3 Department of Obstetrics and Gynaecology, Faculty of Medicine and Health Sciences, Universiti Putra Malaysia, 43400 UPM Serdang, Selangor DE, Malaysia; 4 Bioinformatics Division, The Walter and Eliza Hall Institute of Medical Research, Royal Parade, Parkville, Victoria 3052, Australia; 5 School of Molecular and Biomedical Science, Faculty of Sciences, University of Adelaide, Adelaide, SA 5005, Australia; 6 Department of Human Anatomy, Faculty of Medicine and Health Sciences, Universiti Putra Malaysia, 43400 UPM Serdang, Selangor DE, Malaysia; 7 Department of Molecular Pathology, The Institute of Medical and Veterinary Science and The Hanson Institute, Adelaide, SA 5000, Australia; 8 Howard Florey Institute, The University of Melbourne, Parkville, Victoria 3010, Australia; 9 Current address: Pathology Department, The Peter MacCallum Cancer Centre, St Andrews Place, East Melbourne, Victoria 3002, Australia; 10 Current address: Department of Medical Statistics (Biostatistics), University of Göttingen, Humboldtalle 32, 37073 Göttingen, Germany; 11 Current address: The Bioinformatics Unit, Murdoch Childrens Research Institute, Royal Children's Hospital, Melbourne, Victoria 3052, Australia

## Abstract

**Background:**

Development of the cerebral cortex requires highly specific spatio-temporal regulation of gene expression. It is proposed that transcriptome profiling of the cerebral cortex at various developmental time points or regions will reveal candidate genes and associated molecular pathways involved in cerebral corticogenesis.

**Results:**

Serial analysis of gene expression (SAGE) libraries were constructed from C57BL/6 mouse cerebral cortices of age embryonic day (E) 15.5, E17.5, postnatal day (P) 1.5 and 4 to 6 months. Hierarchical clustering analysis of 561 differentially expressed transcripts showed regionalized, stage-specific and co-regulated expression profiles. SAGE expression profiles of 70 differentially expressed transcripts were validated using quantitative RT-PCR assays. Ingenuity pathway analyses of validated differentially expressed transcripts demonstrated that these transcripts possess distinctive functional properties related to various stages of cerebral corticogenesis and human neurological disorders. Genomic clustering analysis of the differentially expressed transcripts identified two highly transcribed genomic loci, *Sox4 *and *Sox11*, during embryonic cerebral corticogenesis. These loci feature unusual overlapping sense and antisense transcripts with alternative polyadenylation sites and differential expression. The *Sox4 *and *Sox11 *antisense transcripts were highly expressed in the brain compared to other mouse organs and are differentially expressed in both the proliferating and differentiating neural stem/progenitor cells and P19 (embryonal carcinoma) cells.

**Conclusions:**

We report validated gene expression profiles that have implications for understanding the associations between differentially expressed transcripts, novel targets and related disorders pertaining to cerebral corticogenesis. The study reports, for the first time, spatio-temporally regulated *Sox4 *and *Sox11 *antisense transcripts in the brain, neural stem/progenitor cells and P19 cells, suggesting they have an important role in cerebral corticogenesis and neuronal/glial cell differentiation.

## Abstract 2

SAGE analysis reveals spatiotemporally regulated transcripts and overlapping sense and antisense transcripts that are important for mouse cerebral cortex development

## Background

Complex behavioral tasks, from perception of sensory input and the control of motor output to cognitive functions such as learning and memory, are dependent on the precise development of innumerable interconnections of neuronal networks in the cerebral cortex. The development of the cerebral cortex (also known as cerebral corticogenesis) involves the specific influence of intrinsic and extrinsic mechanisms, which are triggered spatio-temporally [1-4]. Between embryonic day 11 (E11) and 18 (E18), the mouse cerebral cortex develops from a relatively homogenous band of mitotic multipotent progenitor cells into a complex laminated structure containing various classes of neuronal cells [2,4-6]. Cerebral corticogenesis involves: proliferation of multipotent progenitors (E11 to E16.5); migration of postmitotic cells (E11 to E17); cell morphogenesis (E13 to E18); gliogenesis and synaptogenesis (E16 until early postnatal period); and reorganization, elimination and stabilization of neuronal networks (up to adulthood).

The mouse cerebral cortex develops in the latero-medial and rostro-caudal axes [7,8]. At E11, the primordial plexiform layer begins to form in the most lateral part of the neural wall. Its growth continues in the latero-medial axis to the medial part of the telencephalon by E13. The primordial plexiform layer is also expanded in the rostro-caudal axis. The growth in this axis is always less than the growth in the latero-medial axis. The first wave of migratory neuronal cells form the cortical plate 2 days later after the development of the primordial plexiform layer. These events are followed by the development of the cortical plate into an organization of six distinct layers that forms the adult cerebral cortex. Generally, the rostral-most regions of the adult cerebral cortex consist of areas involved in executive functions and motor coordination, whilst the caudal-most regions consist of areas involved in sensory perception such as visual function. Although distinct functional arealization of the cerebral cortex do not fully apply to rodents, mounting evidence suggesting that regulated arealization exists has been shown in mice involving transcription factors such as empty spiracles homolog 2 (*Emx2*; *Drosophila*) [9,10], paired box gene 6 (*Pax6*) [9], COUP transcription factor 1 (*Coup-tf1*) [11], Sp8 transcription factor (*Sp8*) [12,13], distal-less homeobox 1/2 (*Dlx1/2*) and gastrulation brain homeobox 2 (*Gbx2*) [14]. The extensive cyto-architectural and anatomical changes occurring in a spatio-temporal manner during the peak (E15) and at the end (E17) of embryonic cerebral neurogenesis as well as during early postnatal (P1) corticogenesis through to adulthood involves complex underlying molecular regulatory networks.

Complex molecular regulatory elements are important determinants in both spatial and temporal cerebral corticogenesis. These elements regulate gene expression at the chromatin, DNA or RNA, and protein levels through chromatin packaging or remodeling, histone acetylation and deacetylation, chromatin insulation, DNA methylation, post-transcriptional regulation and post-translational protein modification or degradation signaling pathways [15-17]. Other processes involved in such regulation include pre-mRNA processing and nuclear mRNA retention by nuclear-specific paraspeckle complexes [18,19], microRNAs (miRNAs) that interfere with mRNA translation [20-22], and a new class of under-characterized non-coding RNA transcripts known as natural antisense transcripts (NATs) [23,24]. These regulatory networks play a pivotal role in establishing when, where and how a multipotent progenitor cell should proliferate, migrate and settle at a designated position in the cortex. The information regarding regulatory networks during cerebral corticogenesis, however, remains incomplete and does not provide a comprehensive view of the underlying regulatory elements throughout this complex event.

In this study, we employed both short and long 3' serial analysis of gene expression (SAGE) technologies [25,26] to identify differentially expressed regulatory elements by comparing transcriptomes of cerebral cortices generated from four selected developmental stages: E15.5, E17.5, P1.5 and 4 to 6 months old. We also compared rostral to caudal regions of E15.5 and left to right regions of adult cerebral cortices. We report temporally co-regulated gene clusters, novel molecular networks and associated pathways, novel candidates in regionalized development and genomic clustering of SRY-box containing gene 4 (*Sox4*) and SRY-box containing gene 11 (*Sox11*) sense and antisense transcripts. The genomic clustering analysis led us to the discovery of spatio-temporal regulation of novel *Sox4 *and *Sox11 *antisense transcripts as well as differential regulation of these transcripts in proliferating and differentiating neural stem/progenitor cells (NSPCs) and P19 (embryonal carcinoma) cells.

## Results

### Generation and analysis of SAGE tags

We constructed 10 SAGE libraries from the cerebral cortex of E15.5, E17.5 and 4- to 6-month-old adult C57BL/6 mice (N = 10; Table 1). The data from two additional SAGE libraries generated from E15.5 and P1.5 cerebral cortices from Gunnersen *et al. *[27] were also incorporated into our analysis. These SAGE libraries represent key stages of cerebral corticogenesis and are accessible from the Gene Expression Omnibus (GEO) website [GEO: GSE15031] [28]. The libraries contain a total of 531,266 SAGE tags (approximately 44,000 tags per SAGE library), 223,471 of which are unique (non-redundant) after screening for artifacts and mapping of short SAGE tags to long SAGE tags (Table 1). The number of unique tags in each library ranges from approximately 7,200 to 32,000 due to the variation in library size (approximately 13,500 to 70,000). The distribution of tag abundance, however, is similar in all libraries (Figures S1 and S2 in Additional data file 1), in which the majority of tags were detected only once (58 to 76% or approximately 5,500 to approximately 24,000 tags), representing a trend comparable with previously reported SAGE analyses of mouse neocortices [27,29]. Of all unique tags, only 5,199 (approximately 2.4%) are common to all developmental stages. The low number of common unique tags is most probably due to the high abundance of tags that occur only once.

**Table 1:** SAGE library information

SAGE library*	Sex	Age	Tissue	Generated sequences	Generated SAGE tag (library)	Unique tags	Unique tags (after scaling to 100,000 tags/library)	GEO accession number
E15_1^†^	U	E15.5	Rostral cerebral cortex	2,044	43,327	15,664	36,153	GSM375449
E15_2^†^	U	E15.5	Caudal cerebral cortex	2,925	39,314	19,929	50,692	GSM375450
E15_3^†‡^	U	E15.5	Cerebral cortex	1,920	44,332	15,787	35,611	GSM375451
E17_1^†^	U	E17.5	Cerebral cortex	384	13,573	7,214	53,150	GSM375638
E17_2^†^	U	E17.5	Cerebral cortex	1,053	47,733	19,508	40,869	GSM375639
P1_1^†‡^	U	P1.5	Cerebral cortex	4,194	42,869	20,465	47,738	GSM375640
Ad_1	M	4 months	Left cerebral cortex	2,016	50,760	19,032	37,494	GSM375458
Ad_2	M	4 months	Left cerebral cortex	1,536	52,476	19,157	36,506	GSM375459
Ad_3	F	5-6 months	Left cerebral cortex	2,688	30,914	15,998	51,750	GSM375460
Ad_4	F	5 months	Left cerebral cortex	1,728	43,592	17,262	39,599	GSM375461
Ad_5	F	5-6 months	Right cerebral cortex	2,684	53,292	21,693	40,706	GSM375462
Ad_6	F	6 months	Right cerebral cortex	3,264	69,084	31,762	45,976	GSM375463

*Each library was constructed by using an independent mouse cerebral cortex. ^†^Short tags were generated from these libraries. ^‡^These libraries were obtained from [27]. F: female; M: male; U: undetermined sex.

### Analysis of differentially expressed transcripts/tags

To identify differentially expressed tags/transcripts (DETs), we considered only those 25,165 unique tags with a count >2 across all libraries. Under stringent analyses (Table S1 in Additional data file 1), we identified a total of 561 DETs in various comparisons between developmental stages (Table 2; Figure S3 in Additional data file 1). A full list of DETs with assigned IDs is provided in Additional data file 2. Greater numbers of DETs are observed when the interval of two comparative developmental stages becomes wider. We find the largest number of DETs (approximately 59% or 328 DETs) in the embryonic versus adult stages (E versus Ad) followed by P1.5 versus Ad (approximately 34% or 192 DETs), E15.5 versus P1.5 (approximately 6% or 36 DETs) and E15.5 versus E17.5 (approximately 7% or 38 DETs) comparisons. These indicate distinctive transcript signatures during cerebral cortex development. Comparisons between rostral and caudal E15.5 (R versus C), and left and right adult cerebral cortices (L versus Ri) are described in a different section below.

**Table 2:** Summary of tag classification into various categories and comparisons

		DETs in various comparisons*						
		
Category	Number of DETs^†^	E15.5 R versus C	Adult L versus Ri	E15.5 versus E17.5	E15.5 versus P1.5	P1.5 versus Ad	E versus Ad	Total
Gene	386 (68.8)	25 (56.8)	10 (58.8)	24 (63.2)	16 (44.4)	114 (59.4)	253 (77.1)	442
EST	33 (5.9)	2 (4.5)	2 (11.8)	1 (2.6)	1 (2.8)	9 (4.7)	24 (7.3)	39
Multiple matches	55 (9.8)	9 (20.5)	3 (17.6)	5 (13.2)	4 (11.1)	21(10.9)	23 (7.0)	65
Ambiguous	44 (7.8)	4 (9.1)	2 (11.8)	6 (15.8)	4 (11.1)	14 (7.3)	20 (6.1)	50
No match	43 (7.7)	4 (9.1)	0 (0)	2 (5.3)	11 (30.6)	34 (17.7)	8 (2.4)	59
Total	561	44	17	38	36	192	328	655

Tag classification into various categories and comparisons was based on the mouse genome assembly released in July 2007. Values in parentheses represent the 'percentage' across a column. *Total number of tags for various comparisons is 655 (rather than 561) due to the same tag being statistically significant in more than one comparison. ^†^Differentially expressed tags. Ad: adult stage; C: caudal region; E: embryonic day/stage; L: adult left hemisphere; P: postnatal days; R: rostral region; Ri: adult right hemisphere.

Approximately 69% of DETs have been assigned to known genes and 6% to expressed sequence tags (ESTs), while 10% of DETs have multiple matches (tag matching multiple gene identifiers) and 8% having ambiguous matches (tag matching the same gene identifier at multiple chromosome loci). Approximately 8% of DETs have no matches and it is most likely that these DETs belong to transcripts from less defined regions, such as centromeric and telomeric areas of a chromosome or assembly gaps of the mouse genome.

### Hierarchical clustering of DETs

**Figure 1:**
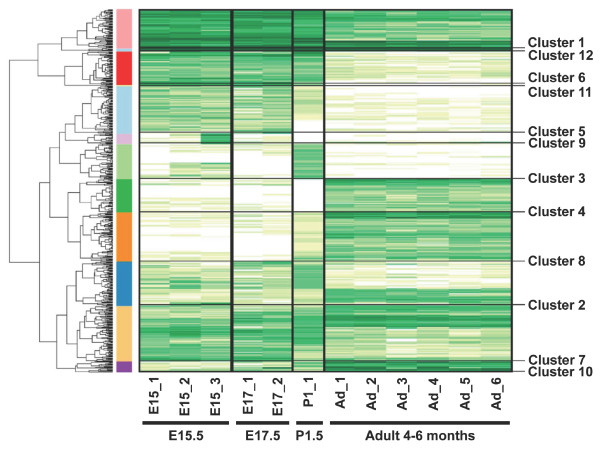
**Hierarchical clustering of 561 differentially expressed transcripts/tags**. Log2 of normalized counts of DETs from cerebral cortices of various developmental stages were clustered. Dark green clusters denote high levels of expression whereas light green to white clusters denote low levels of expression. The x-axis represents the SAGE libraries whereas the y-axis represents the SAGE tags. The panel on the right shows the 12 different clusters.

To identify co-regulated genes, all 561 DETs were hierarchically grouped into 12 clusters based on the calculation of the Euclidean distance of logged normalized counts (Figure 1). Clusters 1, 5 and 6 consist of embryonic-specific DETs that exhibit the highest expression during embryonic development of the cerebral cortex. DETs in cluster 1 are expressed throughout all stages of development but exhibit the lowest expression in the adult cortex. Expression of DETs in cluster 5 ceases prior to birth, whereas DETs in cluster 6 are expressed up to early postnatal stage. On the other hand, clusters 4, 8 and 10 consist of adult specific DETs, showing very similar temporal expression profiles, but with different magnitudes (for example, highest expression in cluster 10). Clusters 2 and 7 are termed 'gene-switching' clusters as they show interesting expression-switching profiles. Cluster 2 shows an expression switch between P1.5 and adult stages whilst cluster 7 shows an expression switch between embryonic and adult stages. Clusters 3 and 9 consist of DETs showing region- (caudal region of E15.5 cerebral cortex) and stage-specific (P1.5 only) expression. Clusters 11 and 12 were excluded from subsequent analyses because they contained very few annotated tags. DETs within the same cluster may be co-regulated and/or involved in similar biological functions during cerebral corticogenesis.

We performed a systematic gene ontology functional clustering using the standardized Gene Ontology term analysis tool DAVID (Database for Annotation, Visualization and Integrated Discovery) [30] (Additional data file 3). Functional analysis of these gene clusters reveals that they have distinctive roles during cerebral corticogenesis. Embryonic-specific gene clusters (1, 5 and 6) are dominated by genes associated with cellular protein and macromolecule metabolic processes or biosynthesis, and nervous system and neuron development. These results match with the expected functional ontologies during embryonic cerebral cortex development in which neuronal migration, differentiation and axonogenesis events are at their peak. In contrast, adult-specific gene clusters (4, 8 and 10) consist of genes related to biological processes in the mature cerebral cortex, such as ion homeostasis, synaptic transmission and regulation of neurotransmitter level. In addition, these gene clusters are also enriched for ribonucleotide/nucleotide binding activity and components of cytoplasmic membrane-bound vesicles. These functional ontologies are in accordance with adult cerebral cortex function, which features synaptogenesis and nerve impulse transmission at synapses. Gene-switching clusters 2 and 7 are enriched with gene ontologies that are similar to both the embryonic- and adult-specific gene clusters. In addition, these gene clusters are also enriched for microtubule cytoskeleton organization and biogenesis, nucleotide biosynthesis and regulation of mRNA translation processes.

### Quantitative RT-PCR validation of DETs and gene clusters

**Figure 2:**
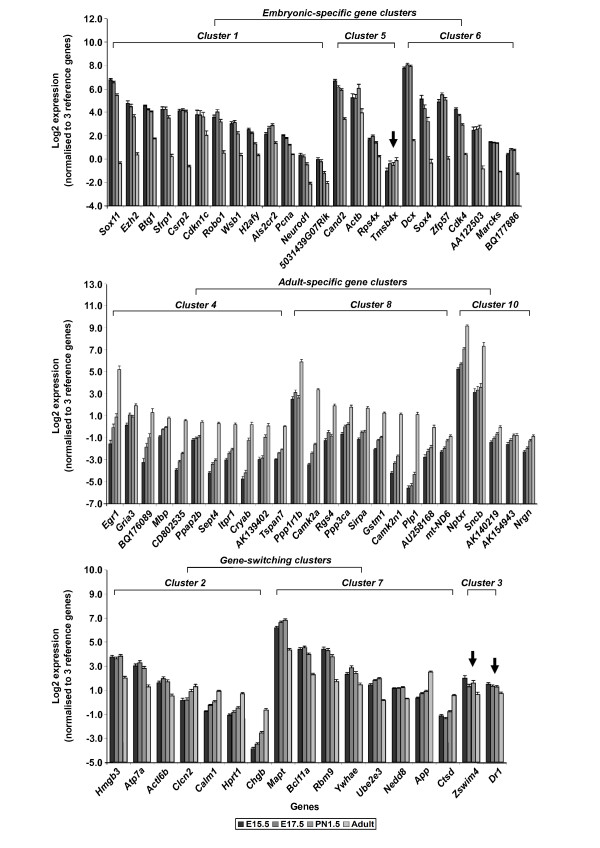
**High-throughput RT-qPCR validation of gene clusters**. All validations were based on DETs for canonical mRNA. Failed validation of DETs according to hierarchical clustering expression profiles is indicated by arrows. N = 3 and data are presented as mean ± standard error of the mean.

To ascertain the robustness of the SAGE datasets, we selected 136 candidate DETs and two additional genes of interests (ATPase, Cu++ transporting, alpha polypeptide, *Atp7a*, and cullin-associated and neddylation-dissociated 2, *Cand2*) for validation after considering both stage-to-stage and hierarchical based analyses (Table S2 in Additional data file 1). The selected DETs are transcription regulators, chromatin modifiers or post-translational regulators, such as ubiquitination pathway related molecules. Seventeen DETs are ESTs, which have been identified in brain-related mouse cDNA libraries or transcriptomes. Independent quantitative RT-PCR (RT-qPCR) validation was carried out using three biological replicates of unpooled cerebral cortex total RNA for each developmental stage. We validated 70 DETs (including 10 ESTs) from SAGE profiles of comparisons between two developmental stages (Additional data file 3) after considering various stringent criteria and cutoffs. The RT-qPCR results for all the 70 validated DETs, *Atp7a *and *Cand2 *are presented in Tables 3, 4, 5 and 6 (Additional data file 4). To validate the expression profiles of gene clusters from the hierarchical clustering analysis, we performed additional RT-qPCR analyses on 65 validated DETs (based on the analysis of two developmental stages), *Atp7a *and *Cand2 *by including other developmental stages (Additional data file 5). The analysis validated 62 out of 65 DETs (Figure 2). Of these, 22 are from embryonic gene clusters, 26 from adult gene clusters, and 14 from gene-switching clusters. We assigned *Atp7a *and *Cand2 *to clusters 2 and 5, respectively, based on their RT-qPCR expression profiles across all the developmental stages. See Tables 3, 4, 5 and 6 for complete list of DETs and their full gene names.

**Table 3:** RT-qPCR validation of SAGE profile for E versus adult comparison

			Fold change			
			
SAGE tag	RefSeq accession	Gene ID	Ad/E15.5 (SAGE)	Ad/E17.5 (SAGE)	Ad/E15.5 (RT-qPCR)	Ad/E17.5 (RT-qPCR)
gcttccccacccccctt	NM_177407	calcium/calmodulin-dependent protein kinase II alpha, *Camk2a*	*As*	*As*	111.59	54.74
ggatatgtggtgtgtac	NM_007913	early growth response 1, *Egr1*	*As*	*As*	108.47	38.93
aaattattgggaaatcc	NM_011123	proteolipid protein (myelin) 1, *Plp1*	28.77	*As*	103.15	89.13
gtatttgcaaaaaaaaa	NM_025451	calcium/calmodulin-dependent protein kinase II inhibitor 1, *Camk2n1*	69.59	59.76	40.08	21.46
gcttcatctccagggag	NM_009964	crystallin, alpha B, *Cryab*	*As*	11.59	30.47	20.58
ttaccatactgggttgg	NM_022029	neurogranin, *Nrgn*	24.67	8.47	43.67	17.00
cctcatttcccctgttc	BQ176089	EST from adult C57BL/6 subfornical organ and postrema tissues	*As*	*As*	23.01	8.91
acccggctagtagtgaa	NM_011129	septin 4, *Sept4*	18.97	16.29	22.38	12.96
ctcattataatcaagaa	CD802535	EST from 27-32 days C57BL/6 hippocampus tissue	*As*	9.05	22.03	13.25
aataaagccaatctgac	NM_033610	synuclein, beta, *Sncb*	20.89	7.66	18.20	15.83
gcttttgttaccatctc	NM_030689	neuronal pentraxin receptor, *Nptxr*	17.88	12.24	15.02	10.93
tccctcccttagtatcc	NM_144828	protein phosphatase 1, regulatory (inhibitor) subunit 1B, *Ppp1r1b*	*As*	*As*	10.47	6.88
gccccttcttcattggc	NM_010358	glutathione S-transferase, mu 1, *Gstm1*	*As*	*As*	9.87	5.58
tgactagcgtgacctgt	NM_007694	chromogranin B, *Chgb*	6.13	6.30	9.43	7.22
atttcttttctggatgg	NM_010585	inositol 1,4,5-triphosphate receptor 1, *Itpr1*	14.34	6.16	9.29	6.12
actttgagattgtacct	NM_009062	regulator of G-protein signaling 4, *Rgs4*	12.23	21.01	8.84	5.32
aataattagccttaggt	AK139402	Mus musculus 10 days neonate cortex cDNA	*As*	*As*	8.28	7.55
ctagacagaggcattat	NM_019634	tetraspanin 7, *Tspan7*	13.08	5.61	7.99	5.38
tgtatacacacacgggt	NM_007547	signal-regulatory protein alpha, *Sirpa*	*As*	*As*	7.18	4.63
tgacaagacactgtggc	AU258168	EST from mouse brain	*As*	*As*	6.49	4.58
cttacctcaggtttcct	NM_008913	protein phosphatase 3, catalytic subunit, alpha isoform, *Ppp3ca*	*As*	5.43	5.48	3.45
atagctttctacacact	NM_007471	amyloid beta (A4) precursor protein, *App*	3.98	2.74	4.56	3.49
tttcagcagtgttggct	NM_013556	hypoxanthine guanine phosphoribosyl transferase 1, *Hprt1*	6.90	8.87	3.45	2.85
aggtatgtacaaagttt	NM_016886	glutamate receptor, ionotropic, AMPA3 (alpha 3), *Gria3*	4.97	8.51	3.40	1.81*
tccaacttgtaactata	NM_009790	calmodulin 1, *Calm1*	4.23	3.17	3.24	2.27
cctcagcctggggtaga	NM_009983	cathepsin D, *Ctsd*	3.46	2.04	3.22	3.84
gcttcgtccacacagcg	NM_010777	myelin basic protein, *Mbp*	202.46	173.85	3.19	2.05
tattaaatgtgcttttt	NM_080555	phosphatidic acid phosphatase type 2B, *Ppap2b*	5.71	2.44	3.02	2.76
cttatcctcacctcagc	NC_005089	NADH dehydrogenase 6, mitochondrial, *mt-ND6*	*As*	*As*	2.65	2.13
caaacctccaaaaacca	AK140219	Mus musculus adult male corpora quadrigemina cDNA	29.22	18.62	2.63	2.03
agtggctaattaggtgt	NM_009900	chloride channel 2, *Clcn2*	14.55	4.16	2.23	2.18
accaatgaacaaaaaaa	AK154943	Mus musculus NOD-derived CD11c +ve dendritic cells cDNA	109.40	51.13	1.76*	1.35 (NS)
ccagtacctgaaaaaaa	NM_008453	Kruppel-like factor 3 (basic), *Klf3*	*As*	-52.97	-1.47^†‡^	-1.27 (NS)
aagaaaacatttaaata	NM_012010	eukaryotic translation initiation factor 2, subunit 3, structural gene X-linked, *Eif2s3x*	-7.70	-10.38	-1.72*	-1.74*
caccctgtgggagctca	NM_172656	amyotrophic lateral sclerosis 2 (juvenile) chromosome region, candidate 2 (human), *Als2cr2*	-11.03	-12.88	-1.75*	-2.48
cctccatcctttatact	NM_009536	tyrosine 3-monooxygenase/tryptophan 5-monooxygenase activation protein, epsilon polypeptide, *Ywhae*	-3.19	-2.52	-1.82*	-2.66
tgtgcttccctgtctta	NM_008683	neural precursor cell expressed, developmentally down-regulated gene 8, *Nedd8*	-4.73	-8.41	-1.83	-1.86
ctcctgaaggcatagtt	NM_009454	ubiquitin-conjugating enzyme E2E 3, UBC4/5 homolog (yeast), *Ube2e3*	-4.14	-5.52	-2.51	-3.22
gtgaaactaaaaaaaaa	NM_009094	ribosomal protein S4, X-linked, *Rps4x*	-10.46	-19.19	-2.76	-3.35
aatgtttctgctttaca	NM_011045	proliferating cell nuclear antigen, *Pcna*	-14.21	-8.30	-3.09	-2.62
cgtggatccctctgtca	NM_009876	cyclin-dependent kinase inhibitor 1C (P57), *Cdkn1c*	-13.68	-7.16	-3.39*	-3.30*
cctttgtgacagtggcc	NM_025635	ZW10 interactor, *Zwint*	7.29	8.84	-3.47^‡^	-4.80^‡^
gaagccagtgggccatc	NM_001033273	RIKEN cDNA 5031439G07 gene, *5031439G07Rik*	-9.10	-10.00	-4.13	-3.65
gctgtgggtcgctgtgg	NM_010561	interleukin enhancer binding factor 3, *Ilf3*	*As*	*As*	-4.15^‡^	-2.97^‡^
acccctgaccccttgtt	NM_016707	B-cell CLL/lymphoma 11A (zinc finger protein), *Bcl11a*	-7.88	-5.52	-4.42	-4.78
cggtgtcccccacctcc	NM_012015	H2A histone family, member Y, *H2afy*	-21.48	-27.88	-4.62	-3.70
cagttgcaataaaaata	NM_010894	neurogenic differentiation 1, *Neurod1*	-7.54	-4.96	-5.62	-5.10
aagtttgcaagtctcca	NM_008538	myristoylated alanine rich protein kinase C substrate, *Marcks*	-24.87	-11.76	-5.72	-5.56
ttgctggcttttataaa	NM_053104	RNA binding motif protein 9, *Rbm9*	-6.10	-7.82	-6.50	-5.97
ggttttgtttgtttgac	NM_019653	WD repeat and SOCS box-containing 1, *Wsb1*	-7.88	-11.96	-6.54	-7.00
tatattgattgtggcaa	NM_007569	B-cell translocation gene 1, anti-proliferative, *Btg1*	-15.28	-16.48	-6.93	-5.58
taagaaacct	NM_019413	roundabout homolog 1 (Drosophila), *Robo1*	-28.64	-16.73	-8.23	-11.35
gctttgactgttctctt	AA122503	EST from M2 cells of skin melanoma	-17.17	-18.68	-9.88	-10.15
tggagcgttggctgtat	NM_009870	cyclin-dependent kinase 4, *Cdk4*	-114.55	-80.88	-14.77	-10.00
ctttccctgccaatgta	NM_013834	secreted frizzled-related protein 1, *Sfrp1*	*Es*	*Es*	-16.48	-16.37
tgcagctttctgttcaa	NM_007971	enhancer of zeste homolog 2 (Drosophila), *Ezh2*	-10.63	-5.52	-21.36	-17.59
cacgacaccccccaccc	NM_009559	zinc finger protein 57, *Zfp57*	-30.72	-42.33	-29.13	-44.46
tgtgtgaggtgttgtga	NM_010025	doublecortin, *Dcx*	-44.41	-58.81	-73.76	-91.10
cagagtgtagtgtgttg	NM_009234	SRY-box containing gene 11, *Sox11*	*Es*	*Es*	-140.05	-120.35

All RT-qPCR data are statistically significant at *P *< 0.001 unless specified: * *P *< 0.01; ^†^*P *< 0.05. ^‡^A disagreement between RT-qPCR and SAGE data. *As*, adult-specific expression; *Es*, embryonic-specific expression; NS, no statistically significant difference between two developmental stages. Fold change values of <1.0 are presented in a negative fold change format.

**Table 4:** RT-qPCR validation of SAGE profile for P1.5 versus adult stages

			Fold change	
			
SAGE tag	RefSeq accession	Gene ID	Adult/P1.5 (SAGE)	Adult/P1.5 (RT-qPCR)
aaattattgggaaatcc	NM_011123	proteolipid protein (myelin) 1, *Plp1*	38.9	43.33
tttcagcagtgttggct	NM_013556	hypoxanthine guanine phosphoribosyl transferase 1, *Hprt1*	6.99	2.26
actcggagccaccagac	NM_009790	calmodulin 1, *Calm1*	4.57	1.83
gcttcgtccacacagcg	NM_010777	myelin basic protein, *Mbp*	*As*	1.79
tccccgtcat	NM_026106	down-regulator of transcription 1, *Dr1*	*Ps*	-1.46
gggaaactaagggagag	NM_172503	zinc finger, SWIM domain containing 4, *Zswim4*	-42.42	-1.96*
gaacgcaagttcagccc	NM_031404	actin-like 6B, *Actl6b*	-5.51	-2.26
gtgaaactaaaaaaaaa	NM_009094	ribosomal protein S4, X-linked, *Rps4x*	-4.72	-2.27
*Nil*	NM_009726	ATPase, Cu++ transporting, alpha polypeptide, *Atp7a*	*Nil*	-2.91
agaagtgtttggagttt	NM_008253	high mobility group box 3, *Hmgb3*	-20.99	-3.53
cctttgtgacagtggcc	NM_025635	ZW10 interactor, *Zwint*	4.65	-4.04^†^
acagtctatgttggagg	BQ177886/NM_010487	C57BL/6 whole brain E15.5 (or known as embryonic lethal, abnormal vision, Drosophila-like 3 (Hu antigen C), *Elavl3*)	-41.99	-4.06
gatacttggaatgacta	NM_007393	actin, beta, cytoplasmic, *Actb*	-18.49	-4.35
ctggcttctt	NM_008538	myristoylated alanine rich protein kinase C substrate, *Marcks*	*Ps*	-4.76
*Nil*	NM_025958	cullin-associated and neddylation-dissociated 2 (putative), *Cand2*	*Nil*	-5.62
gctttgactgttctctt	NM_009870	cyclin-dependent kinase 4, *Cdk4*	-56.55	-5.77
ctcagtaatg	AA122503	EST from M2 cells of skin melanoma	-12.28	-11.12
tggagcgttggctgtat	NM_009238	SRY-box containing gene 4, *Sox4*	-31.51	-11.74
cacgacaccccccaccc	NM_007792	cysteine and glycine-rich protein 2, *Csrp2*	*Ps*	-26.10
tgtgtgaggtgttgtga	NM_009559	zinc finger protein 57, *Zfp57*	-16.33	-32.74
gggacctcgtggaagcc	NM_010025	doublecortin, *Dcx*	-24.56	-82.11

All RT-qPCR data are statistically significant at *P *< 0.001 unless specified: * *P *< 0.01. ^†^A disagreement between RT-qPCR and SAGE data. *As:* adult-specific expression; *Nil*: SAGE data not available; *Ps*: P1.5-specific expression. Fold change values of <1.0 are presented in a negative fold change format.

**Table 5:** RT-qPCR validation of SAGE profile for E15.5 versus P1.5 stages

			Fold change	
			
SAGE tag	RefSeq accession	Gene ID	P1.5/E15.5 (SAGE)	P1.5/E15.5 (RT-qPCR)
Atttctttggtgatttt	NM_010838	microtubule-associated protein tau, *Mapt*	5.51	1.53*
Gcactgttaacaagtgt	NM_009234	SRY-box containing gene 11, *Sox11*	-2.17	-3.32

All RT-qPCR data are statistically significant at *P *< 0.001 unless specified: * *P *< 0.05. Fold change values of <1.0 are presented in a negative fold change format.

**Table 6:** RT-qPCR validation of SAGE profile for rostral E15.5 and caudal E15.5 regions

			Fold change	
			
SAGE tag	RefSeq accession	Gene ID	Caudal/rostral (SAGE)	Caudal/rostral (RT-qPCR)
gttgttcttccagtcgg	NM_016916	bladder cancer associated protein homolog (human), *Blcap*	2.68	1.43
gtcatagctgttctgtg	BC025816	EST sequence BC025816	*Cs*	1.31 (NS)
aagcttgacatttggaa	NM_026187	ankyrin repeat and zinc finger domain containing 1, *Ankzf1*	*Rs*	1.17^† ^(NS)
gatacttggaatgacta	NM_007393	actin, beta, cytoplasmic, *Actb*	-2.63	-1.32
ttggtgaaggaaaaaac	NM_021278	thymosin, beta 4, X chromosome, *Tmsb4x*	-2.33	-2.13 (NS)

All RT-qPCR data are statistically significant at *P *< 0.05 unless specified. ^†^A disagreement between RT-qPCR and SAGE data. *Cs:* caudal region-specific expression; NS: no statistically significant difference between two developmental stages; *Rs:* rostral region-specific expression. Fold change values of <1.0 are presented in a negative fold change format.

According to the comparison between two developmental stages, the most abundantly expressed and validated DETs in the E versus Ad analysis are *Camk2a*, *Egr1 *and *Plp1 *(Table 3). In the adult cerebral cortex, the expression of these DETs is more than 100-fold greater than that at E15.5. Other validated adult-specific DETs with expression levels of approximately tenfold or greater than those in the embryonic brain are *Camk2n1*, *Cryab*, *Nrgn*, BQ176089, CD802535, *Sncb *and *Nptxr*. Conversely, *Sox11*, an embryonic specific DET, is expressed in the E15.5 and E17.5 cerebral cortices with an expression level of at least 100-fold greater than that in the adult. Other validated embryonic specific DETs are *Dcx*, *Zfp57*, *Ezh2*, *Sfrp1 *and *Cdk4*, which have expression levels approximately tenfold or greater than those in the adult.

In the P1.5 versus Ad analysis, *Dcx *is expressed at a level approximately 80-fold greater in P1.5 compared to the adult cerebral cortex (Table 4). Other P1.5 enriched and validated DETs are (in descending order of enrichment) *Zfp57*, *Csrp2*, AA122503, *Cdk4*, *Sox4*, *Marcks*, *Actb*, BQ177889, *Hmgb3*, *Rps4x*, *Actl6b*, *Zswim4 *and *Dr1*, whose expression ranges from 33- to 1.4-fold greater than in the adult. On the other hand, the *Plp1 *is expressed at a level 40 times greater in the adult cerebral cortex compared to P1.5. Other validated genes that are enriched in the adult cerebral cortex include (in descending order) *Hprt1*, *Calm1 *and *Mbp*, with a 2.3- to 1.8-fold greater expression than the P1.5 cerebral cortex. Comparison between E15.5 and P1.5 shows that *Mapt *has a 1.5-fold greater expression level in the P1.5 cerebral cortex while *Sox11 *expression is 3.3-fold lower (Table 5).

We were unable to validate all 17 DETs from L versus Ri regions, suggesting the left and right hemispheres of the adult mouse cerebral cortex are highly similar and indistinguishable at the molecular level. SAGE and RT-qPCR analyses for R versus C regions of E15.5 are discussed in a separate section below.

### Functional analysis of validated gene clusters using Ingenuity Pathway Analysis

The validated DETs of embryonic, adult and gene-switching clusters were functionally characterized using proprietary software, Ingenuity Pathway Analysis (IPA) from Ingenuity Systems^®^, to identify enriched molecular networks and canonical pathways. Given a list of input genes (known as focus genes), IPA mapped these genes to a global molecular network developed from information contained in the Ingenuity knowledge base (a manually curated database of experimentally proven molecular interactions from published literature). Networks of these focus genes were then algorithmically generated based on their connectivity. IPA determined the most significantly enriched biological functions and/or related diseases by calculating the *P*-value using Fisher's exact test. Using similar methods, significantly represented canonical pathways in a set of focus genes were also determined using IPA (Section C in Additional data file 1).

**Figure 3:**
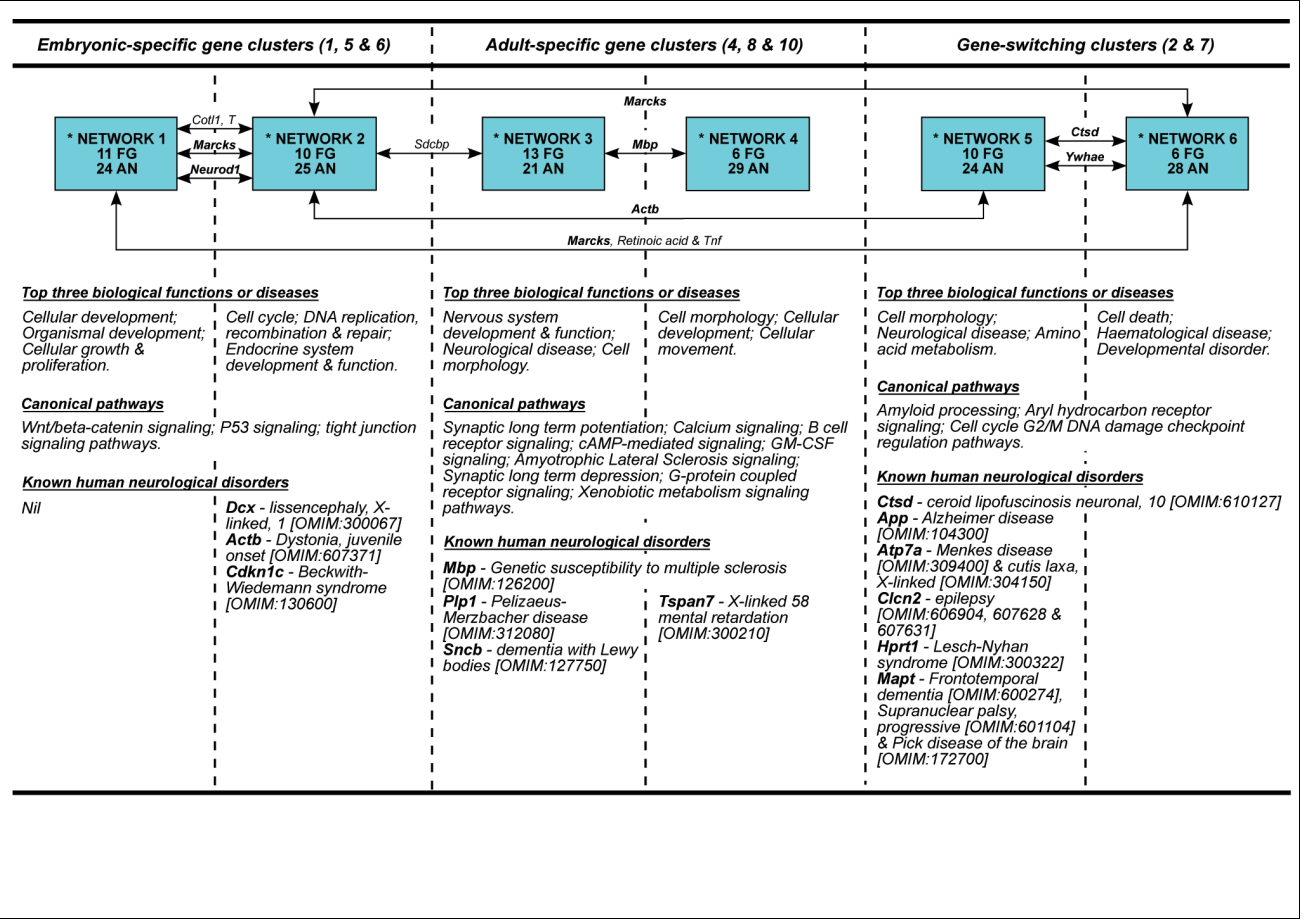
**Novel molecular networks involved in cerebral corticogenesis**. The figure shows novel molecular networks, related biological functions/diseases, canonical pathways and known human neurological disorders based on Ingenuity Pathway Analysis and OMIM database. Detailed molecular interactions for all networks (indicated by asterisks) are illustrated in Figures S4, S5, S6, S7, S8 and S9 in Additional data file 1. Gene names next to arrow lines refer to common genes shared by two networks. Bold gene name refers to a focus gene. AN: associated nodes; FG: focused genes.

From the embryonic-specific gene clusters, we identified two statistically significant molecular networks (made up of 19 focus genes and 47 associated nodes; networks 1 and 2 in Figure 3; Figures S4 and S5 in Additional data file 1). The networks are interconnected through two genes, *Marcks *and *Neurod1*. In general, these networks are associated with cell cycle and DNA replication, recombination and repair processes. Three statistically significant (using *P *< 0.05 as a cutoff) canonical pathways are enriched in these networks (Figure 3); Wnt/β-catenin signaling (*Sox4*, *Sox11 *and *Sfrp1*), P53 signaling (*Cdk4 *and *Pcna*) and tight junction signaling (*Cdk4 *and *Actb*) pathways. Validated DETs such as *Btg1*, *Cdk4*, *Cdkn1c*, *Csrp2*, *Ezh2*, *Neurod1*, *Pcna*, and *Rps4x *are associated with cell cycle control whereas *Actb*, *Ezh2*, *Als2cr2*, *Marcks*, *Robo1 *and *Dcx *are associated with cellular assembly and organization. These processes are important in the formation of filopodia, membrane blebs and growth cones during neuronal growth, migration and axonogenesis. Known human neurological disorders associated with the networks, particularly network 2, include X-linked lissencephaly (Online Mendelian Inheritance in Man [OMIM:300067]; DCX), juvenile onset dystonia ([OMIM:607371]; ACTB) and Beckwith-Wiedemann syndrome ([OMIM:130600]; CDKN1C). All the DETs implicated in these networks are expressed in the cortical plate with the exception of *Pcna *(Table S5 in Additional data file 1) [31-42].

In adult-specific gene clusters, two molecular networks (18 focus genes and 50 associated nodes; networks 3 and 4 in Figure 3) were identified and interconnected via a single gene, *Mbp *(Figures S6 and S7 in Additional data file 1). These molecular networks enrich for nine statistically significant canonical pathways (*P *< 0.05) such as synaptic long-term potentiation and depression, calcium signaling, B cell receptor signaling, cAMP-mediated signaling, GM-CSF signaling, amyotrophic lateral sclerosis signaling, G-protein-coupled receptor signaling and xenobiotic metabolism signaling pathways. Validated DETs such as *Camk2a*, *Gria3*, *Itpr1*, *Egr1*, *Rgs4*, *Gstm1 *and *Ppp3ca *are associated with cell-to-cell communication in the adult cerebral cortex. Biological functions such as cell proliferation, movement and death processes are also linked to these networks through the genes *Camk2a*, *Cryab*, *Egr1*, *Gstm1*, *Itpr1*, *Mbp*, *Nrgn*, *Ppp1r1b*, *Ppp3ca*, *Rgs4*, *Sept4*, *Sirpa *and *Sncb*. Known human neurological disorders associated with Network 3 include Pelizaeus-Merzbacher disease ([OMIM:312080]; PLP1) and dementia with Lewy bodies ([OMIM:127750]; SNCB), whereas X-linked mental retardation ([OMIM:300210]; TSPAN7 or TM4SF2) is associated with network 4. MBP, found in both network 3 and 4, is associated with genetic susceptibility to multiple sclerosis [OMIM:126200]. All the DETs implicated in these networks are expressed in layers I to VI of the cerebral cortex, with the exception of *Gstm1 *(layers II to V), *Sirpa *and *Ppp3ca *(layers II to VI), and *Nptxr *(no expression data available) (Table S5 in Additional data file 1) [32-34,43-52].

Both networks 5 and 6 (Figure 3) are linked to gene-switching clusters. These two molecular networks (14 focus genes and 52 associated nodes) are associated with cellular morphogenesis, amino acid metabolism, cell death processes and developmental disorders (Figures S8 and S9 in Additional data file 1). These networks are connected by *Ctsd *and *Ywhae *and are implicated in amyloid processing (*App *and *Mapt*), aryl hydrocarbon receptor signaling (*Ctsd *and *Nedd8*) and cell cycle G2/M DNA damage checkpoint regulation pathways (*Ywhae*). *App*, *Atp7a*, *Hprt1*, *Mapt*, *Ctsd *and *Clcn2 *are involved in cell morphogenesis, assembly and organization. Among all the networks in the study, network 5 has the greatest number of DETs associated with known human neurological disorders with six out of ten focus genes being associated with known neurological disorders such as neuronal ceroid lipofuscinosis ([OMIM:610127]; CTSD), Alzheimer's disease ([OMIM:104300]; APP), Menkes disease ([OMIM:309400]; ATP7A), X-linked cutis laxa ([OMIM:304150]; ATP7A), epilepsy ([OMIM:606904, 607628 and 607631]; CLCN2), Lesch-Nyhan syndrome ([OMIM:300322]; HPRT1), frontotemporal dementia ([OMIM:600274]; MAPT), progressive supranuclear palsy ([OMIM:601104]; MAPT) and Pick disease of the brain ([OMIM:172700]; MAPT). All the DETs implicated in these networks are expressed in the cortical plate during embryonic development with the exception of *Ube2e3*. In the adult, all DETs are expressed in layers I to VI of the cerebral cortex except for *Bcl11a*, *Clcn2*, *Hprt1 *(layers II to VI), *Atp7a *and *Ube2e3 *(no expression was detected in the adult cerebral cortex) (Table S5 in Additional data file 1) [32-35,46,53-61].

To refine the functional analysis to a cellular level, we grouped all 40 RT-qPCR validated DETs from networks 3, 4, 5 and 6 (adult specific and gene-switching gene clusters) into three groups according to where they are expressed: only in cortical neurons (N group); only in cortical glia (G group); and in both cortical neurons and glia (B group). None of the DETs from networks 1 and 2 (embryonic-specific gene clusters) were analyzed because most of the cells within the cerebral cortex are committed to the neuronal lineage at E15.5. The methods used to tabulate and group validated DETs are detailed in Section D in Additional data file 1. DETs classified as part of the N group are *Bcl11a*, *Calm1*, *Camk2a*, *Camk2n1*, *Hprt1*, *Itpr1*, *Mapt*, *Nedd8*, *Nrgn*, *Ppp1r1b*, *Ppp3ca*, *Rbm9*, *Rgs4*, *Sncb *and *Ywhae*, whereas those in the G group include *Cryab*, *Mbp*, *Nptxr*, *Plp1*, *Ppap2b*, *Sept4 *and *Tspan7*. The B group consists of *App*, *Atp7a*, *Chgb*, *Ctsd*, *Egr1*, *Gria3*, *Gstm1 *and *Sirpa*. DETs without any cellular expression information, such as *Actl6b*, *Clcn2*, *Hmgb3*, *Ube2e3*, AK138272, AK139402, AK140219, AK154943, AU258168 and BQ176089, were placed into group B to facilitate downstream analysis. Of the 40 DETs, only 35 were mapped to the IPA knowledgebase and subjected to further analysis. Functional analysis of these 35 DETs show that the neuron-based DETs (N and B groups) are enriched for various human neurological disorders, such as schizophrenia (9 DETs; *P *= 4.36E-8), Huntington's disease (8 DETs; *P *= 1.03E-5), X-linked mental retardation (4 DETs; *P *= 3.78E-7), Parkinson's disease (3 DETs; *P *= 6.99E-3) and Alzheimer's disease (3 DETs; *P *= 1.52E-2) (Figure S10 in Additional data file 1). Thirteen DETs associated with these neurological disorders are also implicated in processes related to cell death. Of these, eight DETs are expressed in neurons only, two in glia only and three in both neurons and glia.

From the above IPA analysis, *Cand2 *(embryonic gene clusters), *Camk2n1 *(adult gene clusters), *Hmgb3 *(gene-switching clusters) and 10 ESTs (BQ176089, CD802535, AK139402, AU258168, AK138272, AK140219, AK154943, AA122503, NM_001033273 and BQ177886) are not currently connected to any networks.

### Regionalized expression of DETs in the E15.5 cerebral cortex

Early regionalized development is an important event that could lay the foundations for adult arealization of cerebral functions. To identify genes with regionalized expression profiles, we compared SAGE libraries generated from rostral and caudal regions (equivalent to anterior and posterior regions of the human cerebral cortex) of the E15.5 cerebral cortex. We identified 44 DETs and selected 25 DETs (22 known genes, 1 EST and 2 ambiguous genes; Additional data file 2) and 2 genes of interest (bladder cancer associated protein, *Blcap*, and ankyrin repeat and zinc finger domain containing 1, *Ankzf1*) for RT-qPCR validation and further detailed region-based analysis. This was done using independent biological triplicates of clearly defined regions/quadrants, such as rostro-lateral (RL), rostro-medial (RM), caudo-lateral (CL) and caudo-medial (CM) of the E15.5 cerebral cortex (see Materials and methods). Two positive controls with known regionalised expression were included in the RT-qPCR: RAR-related orphan receptor beta (*Rorb*) and nuclear receptor subfamily 2, group F, member 1 (*Nr2f1*). *Rorb *is highly expressed in the rostral region whereas *Nr2f1 *is highly expressed in the caudal region of the cerebral cortex [1,62].

**Figure 4:**
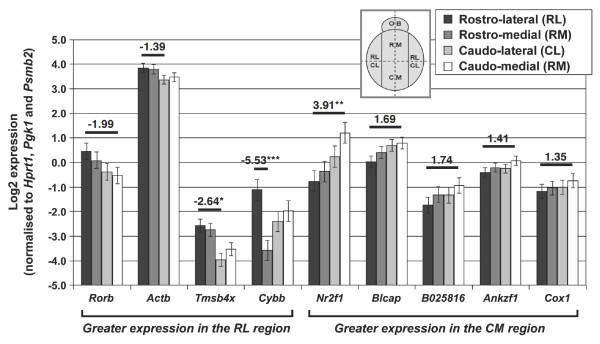
**RT-qPCR analysis of all R versus C DETs based on quadrant versus quadrant analysis**. RL, RM, CL and CM denote rostro-lateral, rostro-medial, caudo-lateral and caudo-medial regions of the cerebral cortex, respectively. OB denotes olfactory bulb. N = 3 per quadrant and data are presented as mean ± standard error of the mean. Fold change values (normalized to RL) are presented above the comparative bar and any values <1 are presented in the negative fold change format. Only the most significant fold change value is presented for each target gene. * P < 0.05; ** P < 0.01; *** P < 0.001.

An initial RT-qPCR analysis of combined RL and RM (rostral) as well as CL and CM (caudal) regions shows upregulation of *Rorb *and *Nr2f1 *in rostral and caudal regions, respectively (based on fold change direction and magnitude of 1.3 times). The same analysis also confirmed the SAGE data for 3 out of 25 DETs (*Actb*, *Tmsb4x *and BC025816) and *Blcap *(Table 6; Additional data file 6). Both *Actb *and *Tmsb4x *have greater expression in the rostral region whereas *Blcap *and BC025816 are greater in the caudal region. To identify expression profiles in a more refined area and prevent regional compensation due to combined quadrant analysis, we performed a quadrant versus quadrant multiple regions comparison. The largest number of DETs were found in the RL versus CM comparison, as they are the two developmentally most distinct regions within the cerebral cortex at E15.5 compared to others; RL versus CL > RM versus CL > RL versus RM = RM versus CM > CL versus CM. The region-specific expression profiles were plotted for each of the DETs (Figure 4) and grouped into two categories: RL-specific DETs, such as *Actb*, *Tmsb4x *and cytochrome b-245, beta polypeptide (*Cybb*); and CM-specific DETs, including *Blcap*, EST BC025816, *Ankzf1 *and cytochrome c oxidase I, mitochondrial (*Cox1*) (Additional data file 6).

**Figure 5:**
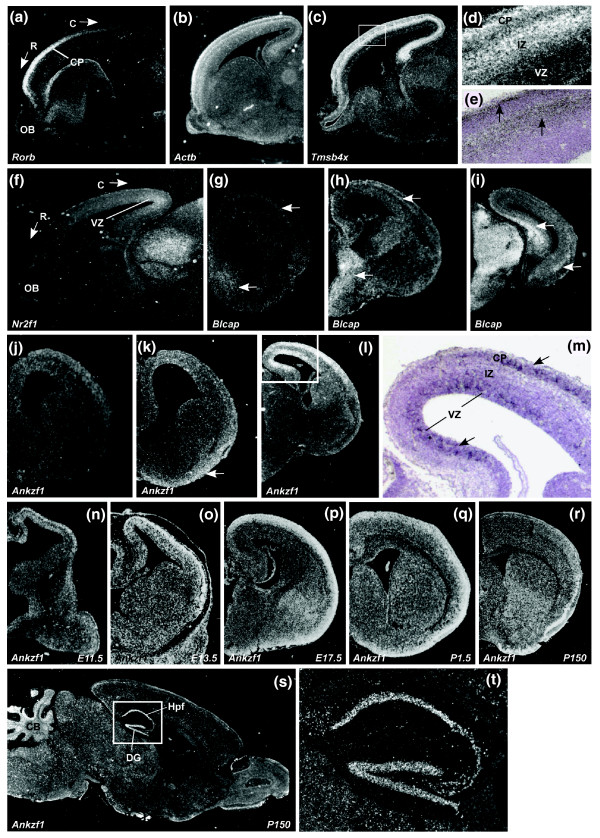
***In situ *RNA hybridization of selected R versus C regions of E15.5 DETs**. ISH was performed on **(a-m) **E15.5 and **(n-t) **E11.5 to P150 brains. (g-m) Coronal sections that are generated from the rostral to caudal axis; (a-f, s-t) sagittal sections. Micrographs of higher magnification are presented directly after any micrographs with an inset box (d-e, m, t). All micrographs are in dark-field except for (e, m), which are bright-field micrographs. C: caudal; CB: cerebellum; CP: cortical plate; DG: dentate gyrus; HPf: hippocampal formation; IZ: intermediate zone; OB: olfactory bulb; R: rostral; VZ: ventricular zone. Arrows show the region with expression or silver grains.

To visualize the regionalized expression profiles, we performed *in situ *RNA hybridization (ISH) on all DETs validated by quadrant versus quadrant RT-qPCR analysis, *Blcap*, *Ankzf1 *as well as the positive controls *Rorb *and *Nr2f1*. We performed ISH on sagittal and coronal sections (from rostral to caudal regions) of the E15.5 mouse brain (Figure 5). Under dark-field microscopic examination, we confirmed the regionalized expression of *Rorb *(at the rostral cortical plate; Figure 5a) and *Nr2f1 *(at the caudal ventricular zone; Figure 5f). From the analysis, *Actb *is highly expressed at the cortical plate and the subplate (Figure 5b). *Tmsb4x *is highly expressed at the cortical plate and the intermediate zone (Figure 5c-e).

ISH analysis showed that both *Blcap *and *Ankzf1 *are caudal specific. Serial coronal sections from rostral to caudal regions of the brain (Figure 5g-i) show *Blcap *is weakly expressed in the rostral cerebral cortex, particularly at the intermediate zone, subplate and the cortical plate, but is highly expressed in the hippocampus and thalamus (Figure 5i). *Ankzf1 *is expressed specifically in the ventricular zone as well as the cortical plate towards the caudal region of the cerebral cortex (Figure 5j-m). The distinctive expression of *Ankzf1 *in both the ventricular zone and cerebral cortex prompted us to extend our ISH analysis to various developmental stages starting from E11.5 to adulthood (Figure 5n-t). *Ankzf1 *is expressed in the primordial plexiform and the ventricular zone layers of the telencephalon at E11.5 (Figure 5n). By E13.5, *Ankzf1 *is weakly expressed in the ventricular zone, but is highly expressed in the cortical plate (Figure 5o). From E17.5 to P1.5, the expression of *Ankzf1 *is maintained in the cerebral cortex (Figure 5p, q). In the adult, *Ankzf1 *expression is obvious in the piriform cortex, hippocampus and cerebellum (Figure 5r-t).

### Genomic clustering of sense-antisense SAGE tags at the *Sox4 *and *Sox11 *loci

We performed genomic clustering analysis of the SAGE tags to determine any actively transcribed chromosomal loci throughout cerebral corticogenesis. Probabilities for chance occurrences of two, three, four, and five DETs being clustered within a window of ten adjacent tags present within each chromosomal location, irrespective of genetic distance, were calculated. This analysis was based on the DET lists described above (Additional data file 2). The analysis showed two overrepresented chromosome loci at *Sox4 *and *Sox11*, which derive from embryonic-specific gene clusters.

**Figure 6:**
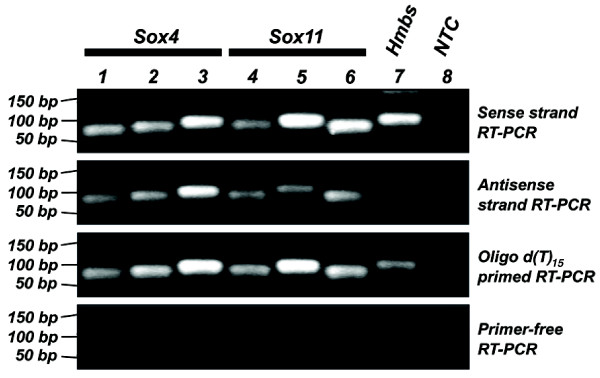
**Strand-specific RT-PCR**. Lanes 1 to 6 represent the amplification of the *Sox4 *and *Sox11 *transcripts using more than one probe. Lane 7 shows the amplification of the *Hmbs *housekeeping gene whereas lane 8 represents the amplification of water as non-template control (NTC) using the primer set for *Hmbs*. The first panel consists of amplicons generated from the reverse-transcribed sense strand cDNA whereas the second panel consists of amplicons generated from the reverse-transcribed antisense strand cDNA. The third panel represents amplicons generated from oligo-d [T]_15 _primed reverse-transcribed cDNA, which serves as a positive control. The last panel represents amplicons generated from primer-free reverse-transcription reactions. The numbers on the left indicate the size of the generated bands.

At both loci, we observed multiple SAGE tags with both sense and antisense orientations, which signify alternative polyadenylation sites, differential splicing and overlapping antisense transcription. As an initial validation of the antisense messages, we performed strand-specific RT-PCR (Figure 6) using cDNA synthesized from equally pooled total RNAs (three mice from each of E15.5, E17.5, P1.5 and adult stages). Three primer sets were used for each locus (designed at the middle, 5'- and 3'-ends of the canonical transcripts). For both *Sox4 *and *Sox11 *loci, the analysis showed positive signals from all primer sets used that were complementary to the antisense strand, therefore confirming the presence of one or more antisense transcript(s) that span the canonical transcript. Hydroxymethylbilane synthase gene (*Hmbs*) served as a negative control and there were no positive bands in the antisense strand RT-PCR, confirming the absence of antisense transcripts.

### Genomic cluster at the *Sox4 *gene locus

**Figure 7:**
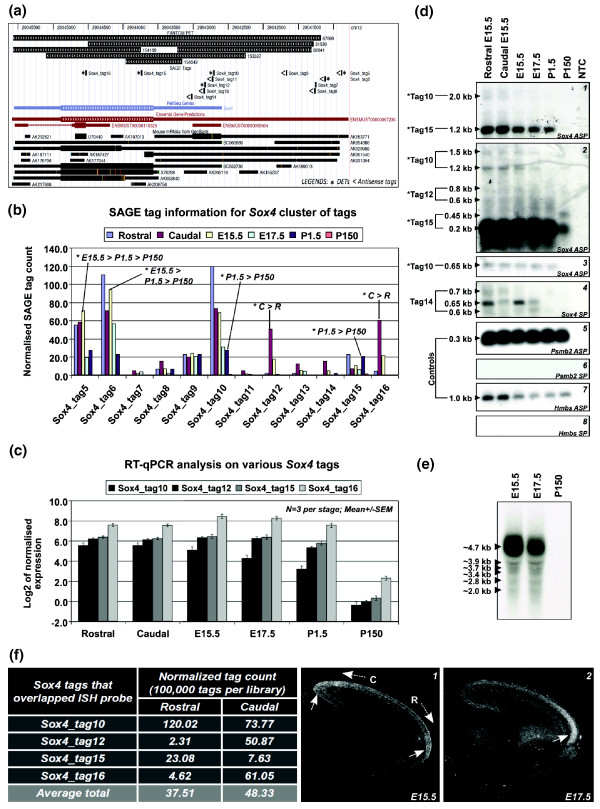
**Genomic cluster at the *Sox4 *locus**. **(a) **The UCSC genome browser of the over-represented *Sox4 *genomic locus. SAGE tags were found in both directions within the *Sox4 *reference gene. **(b) **The SAGE expression profiles for each tag in the *Sox4 *locus. **(c) **The RT-qPCR validations of selected DETs at various loci within the *Sox4 *canonical gene. **(d) **The 3' RACE-Southern blotting analysis. The left panel of the figure shows the sizes of the bands and their corresponding tags. (d1-d3) Amplification of *Sox4 *sense transcripts (ASP); (d4) amplification of *Sox4 *antisense transcripts (SP). Tags with asterisks are DETs. Both (d5) and (d7) are positive controls exclusively generated from the sense strand of *Psmb2 *and *Hmbs *housekeeping genes (ASP), respectively. The corresponding (d6) and (d8) are the antisense expression (negative control) of *Psmb2 *and *Hmbs *(SP), respectively. **(e) **Northern analysis of total RNA isolated from pooled mouse cerebral cortices (N = 7). **(f) **The regionalized expression of *Sox4 *sense transcripts determined by ISH. The table consists of SAGE information for related *Sox4 *sense tags at rostral and caudal regions of the E15.5 cerebral cortex. (f1) and (f2) are sagittal sections obtained from E15.5 and E17.5 mouse brains, respectively. The figures show the expression of the *Sox4 *sense transcript variants. Arrows show brain regions with greater *Sox4 *sense expression. C: caudal region of the cerebral cortex; R: rostral region of the cerebral cortex.

We mapped SAGE tags from the genomic cluster to the *Sox4 *gene locus using the University of California Santa Cruz (UCSC) genome browser (Figure 7a; Additional data file 7) [63]. Only tags within and around the *Sox4 *canonical transcript are shown. Evidence of mapped mouse mRNAs within this locus further justifies the existence of multiple SAGE tags in addition to the canonical *Sox4 *transcript. Subsequent validation of the genomic clusters was solely based on the SAGE tags situated within the canonical transcript. Based on the SAGE tag information, 6 out of 12 tags are DETs. These include four DETs within the canonical transcript (Figure 7b), with sox4_tag10 and sox4_tag15 having greater expression in P1.5 compared to P150 (adult stage), whereas sox4_tag12 and sox4_tag16 are both abundantly expressed in the caudal region of the E15.5 cerebral cortex. Based on the RT-qPCR analysis (Figure 7c), sox4_tag10 has greater expression in E15.5 compared to E17.5 (1.76-fold change), P1.5 (3.72-fold change) and Ad (43.67-fold change). For sox4_tag12, sox4_tag15 and sox4_tag16, differences are seen only in P1.5 (-2.00-fold, -1.57-fold and -1.84-fold changes, respectively) and Ad (-82.08-fold, -68.92-fold and -69.41-fold changes, respectively) when compared to E15.5. RT-qPCR analysis on the same tags did not find any differences between rostral and caudal cerebral cortices of E15.5. The differences in fold change between DETs suggest irregular overlapping of various transcript variants at different *Sox4 *gene loci.

To further validate the expression profiles of the multiple *Sox4 *DETs, we performed 3' rapid amplification of cDNA ends (RACE)-Southern analysis using pooled adaptor-oligo-d [T]_15 _synthesized cDNAs from three mice at each developmental stage. Based on this method, we were able to semi-quantitatively and accurately measure the expression levels of individual SAGE tags at the locus. To show that the amplification was cDNA specific, we performed PCR by using the same primer sets on mouse genomic DNA under the same conditions. In all cases, no amplification was observed (data not shown). This analysis confirmed the presence of four out of seven alternative transcripts for *Sox4 *(Figure 7d). Corresponding tags were determined by estimating the amplicon sizes between the strand-specific primers used, the next downstream AAUAAA/AUUAAA polyadenylation signal (if any) and succeeding CATG sequence or SAGE tags. Figure 7d(1)-d(3) confirms the existence of sox4_tag10, sox4_tag12 and sox4_tag15. Of these tags, SAGE expression profiles of sox4_tag10 and sox4_tag15 were validated (embryonic-specific and reduced expression after P1.5) but not sox4_tag12 (E15.5 caudal region-specific). 3' RACE-Southern analysis using a sense probe detected bands in the rostral and caudal regions of E15.5, E15.5 and E17.5 cerebral cortices and, therefore, confirmed the existence of the *Sox4 *antisense transcripts (Figure 7d(4)). Even though none of these tags were differentially expressed in between these regions based on the SAGE analysis, our findings show distinctive regionalization for sox4_tag14 expression at the E15.5 rostral cerebral cortex. Proteasome (prosome, macropain) subunit, beta type 2 gene (*Psmb2*) and *Hmbs *were used as controls and no antisense or alternative transcripts were identified at these gene loci (Figure 7d(5)-d(8)).

Since 3' RACE-Southern analysis was dependent on oligo- [dT]_15 _priming, we could not rule out the possibility of amplicons that were generated by false priming on homopolymer-A stretches. Therefore, Northern analyses were performed on equally pooled total RNA extracted from the cerebral cortices of seven mice at E15.5, E17.5 and P150 (negative control). By using a double-stranded DNA probe at the 3' untranslated region (UTR) of *Sox4 *(Additional data file 8), we identified six bands ranging from approximately 2 kb to approximately 4.7 kb (Figure 7e). *Sox4 *sense transcripts are weakly expressed in the adult, but highly expressed in the embryonic cerebral cortex. The number of bands observed is similar and corresponds to overlapping mouse mRNAs as well as publicly available paired-end diTag (PET) sequences downloaded from Ensembl (Figure 7a; Table S6 in Additional data file 1) [64]. Taken together, the analysis confirmed the existence of multiple overlapping variants of *Sox4 *sense and antisense transcripts at this gene locus.

To confirm the rostro-caudal expression of *Sox4 *sense transcripts, we performed ISH on sagittal sections of mouse brains using a *Sox4 *antisense riboprobe that spanned across the sox4_tag10, sox4_tag12, sox4_tag15 and sox4_tag16 SAGE tags. *Sox4 *showed regionalized expression at E15.5 and E17.5 (Figure 7f). At E15.5, *Sox4 *sense transcripts are expressed more in both the rostral- and caudal-end regions of the cortical plate compared to the intermediate region between them (Figure 7f(1)). By E17.5, expression of *Sox4 *sense transcripts is obvious in the rostral cortical plate (Figure 7f(2)). At both stages of development, *Sox4 *sense transcripts are uniformly expressed in the intermediate zone of the cerebral cortex. These findings correspond to the SAGE tag counts for E15.5 rostro-caudal regions of the cerebral cortex (Figure 7f). These observations explain the averaged total tag count per 100,000 tags for different *Sox4 *sense transcripts, which are predominantly expressed in both rostral and caudal regions of the cerebral cortex. The regionalized expression of *Sox4 *in the cortical plate is obvious only at E15.5 and E17.5, but not at other stages of development (Figure S11 in Additional data file 1).

### *In situ *RNA hybridization of *Sox4 *sense and antisense transcripts

**Figure 8:**
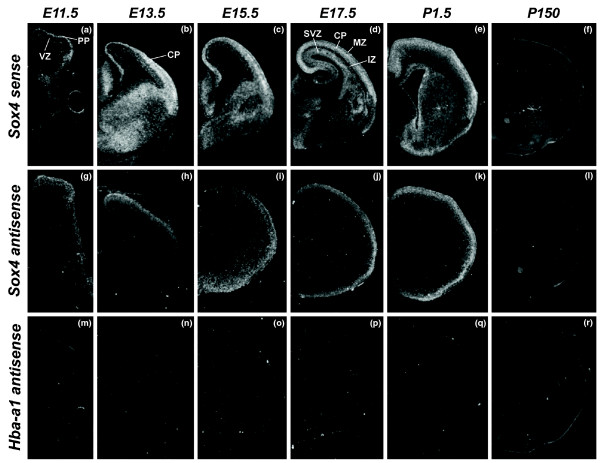
**ISH analysis of *Sox4 *transcripts in E11.5 to P150 mouse brains**. **(a-f) **Expression of the sense transcript for *Sox4*. **(g-l) **The expression of the antisense transcript for *Sox4*. **(m-r) ***Hba-a1 *antisense expression (negative control). All micrographs were taken from coronal sections. CP: cortical plate; IZ: intermediate zone; MZ: marginal zone; PP: primordial plexiform layer; SVZ: subventricular zone; VZ: ventricular zone.

To further ascertain the antisense expression of *Sox4 *in a spatio-temporal manner, we performed ISH on coronal sections obtained from E11.5, E13.5, E15.5, E17.5, P1.5 and P150 mouse brains. Sense and antisense RNA probes were generated from the same clone used in the Northern analysis. At E11.5, *Sox4 *sense transcripts are confined to the primordial plexiform layer (Figure 8a). From E13.5 to P1.5, the sense transcripts are expressed throughout the cortical plate (Figure 8b-e). Expression of sense transcripts in the subventricular zones is observed at E17.5 and P1.5 only (Figure 8d, e). There is no observable sense expression in the adult stage (Figure 8f). *Sox4 *antisense transcripts are expressed throughout the telencephalon at E11.5 (Figure 8g). From E13.5 to P1.5, *Sox4 *antisense expression is confined to the cortical plate only (Figure 8h-k). There is no obvious antisense expression in the cerebral cortex in the adult stage (Figure 8l). A microscopic examination at high magnification showed that *Sox4 *antisense transcripts are predominantly localized in the nucleus whereas *Sox4 *sense transcripts are found in both the nucleus and cytoplasm (Figures S12, S13 and S14 in Additional data file 1). We used hemoglobin alpha, adult chain 1 (*Hba-a1*) of the corresponding brain region and time-point as a control in the analysis (Figure 8m-r).

**Figure 9:**
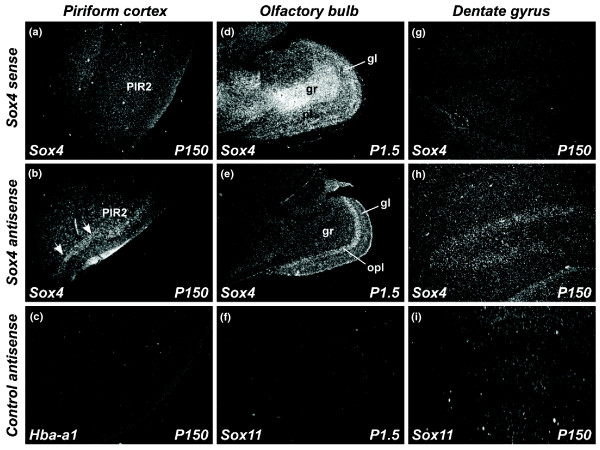
***Sox4 *antisense expression in other brain regions**. **(a-i) ***Sox4 *antisense expression is also observed in other regions such as the piriform cortex (a-c; arrows), olfactory bulb (d-f) and dentate gyrus (g-i). (a, d, g) *Sox4 *sense expression; (b, e, h) *Sox4 *antisense expression. (c, f, i) *Hba-a1 *or *Sox11 *antisense expression (negative controls). All micrographs were taken from sagittal sections except (a-c), which were taken from coronal sections. gl: glomerular layer; gr: granule layer; Opl: outer plexiform layer; PIR2: piriform cortex layer II.

Furthermore, *Sox4 *antisense expression occurs in the piriform cortex layer II (Figure 9a-c) and dentate gyrus (Figure 9g-i) in the adult brain; however, no sense expression is observed in these regions. At P1.5, we identified complementary expression between *Sox4 *sense and antisense transcripts in the olfactory bulb (Figure 9d-f). *Sox4 *sense expression was confined to the granular and glomerular layers of the olfactory bulb whereas antisense expression was found only in the outer plexiform layer. We used either *Sox11 *or *Hba-a1 *of the corresponding brain region and time-point as a control in the analysis (Figure 9c, f, i).

### Analysis of the *Sox11 *genomic cluster

SAGE tags, which represent multiple overlapping sense and antisense transcript variants at the *Sox11 *genomic cluster, were validated using 3' RACE-Southern analysis as described above. See Section F in Additional data file 1 for a full description of the *Sox11 *results. ISH analysis did not confirm the expression of antisense transcripts of *Sox11*, but the presence of PETs spanning three out of five antisense tags confirmed the existence of *Sox11 *antisense transcripts (Table S8 and Figures S16, S17 and S18 in Additional data file 1). The discrepancy between ISH and RT-qPCR or 3' RACE-Southern analysis suggests that *Sox11 *antisense transcripts might be expressed at low levels or at specific locations of the cerebral cortex, and hence can be detectable only by using serial sections or whole mount ISH.

### Screening of *Sox4 *and *Sox11 *antisense transcripts in the adult mouse brain, organs, P19 cell line and neurospheres

**Figure 10:**
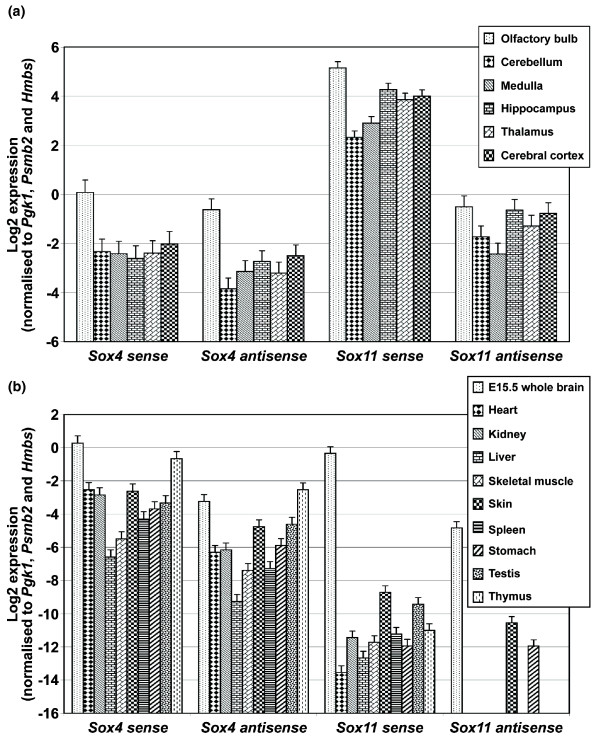
**Expression of *Sox4 *and *Sox11 *transcripts in various mouse organs**. **(a) **Strand-specific RT-qPCR screening of *Sox4 *and *Sox11 *sense and antisense transcript expression in various adult mouse brain regions. N = 2 and data are presented as mean ± standard error of the mean (SEM). **(b) **Strand-specific RT-qPCR screening of *Sox4 *and *Sox11 *sense and antisense transcripts in various adult mouse organs. N = 3 and data are presented as mean ± SEM.

We screened various adult brain regions (olfactory bulb, cerebellum, medulla, hippocampus, thalamus and cerebral cortex) and selected mouse organs (E15.5 whole brain, heart, kidney, liver, skeletal muscle, skin, spleen, stomach, testis and thymus) for the expression of *Sox4 *and *Sox11 *antisense transcripts by strand-specific RT-qPCR. Within the adult brain, *Sox4 *sense and antisense transcripts are expressed in all regions, with the highest level found in the olfactory bulb, which is approximately four- to nine-fold greater than those in other brain regions (Figure 10a). Expression of *Sox4 *antisense transcripts occurs in all mouse organs, with the highest level in the thymus followed by E15.5 whole brain, testis and skin (Figure 10b). *Sox4 *sense and antisense expression profiles are similar throughout the entire series of samples screened, with the sense transcripts being consistently expressed at a greater level than the antisense transcripts (approximately 1.7-fold in various brain regions and approximately 2- to 14-fold in various organ comparisons).

*Sox11 *sense transcripts are expressed at the highest level in the olfactory bulb, approximately two- to seven-fold greater than those in other brain regions (Figure 10a). *Sox11 *antisense transcripts, on the other hand, are expressed in all brain regions screened and at a comparable level in the olfactory bulb, hippocampus, thalamus and cerebral cortex. In comparison to other adult mouse organs, *Sox11 *sense and antisense transcripts are highly expressed in the E15.5 whole brain, with *Sox11 *sense transcript levels at least 100-fold greater than those in other mouse organs (Figure 10b). On the other hand, *Sox11 *antisense expression is observed only in the E15.5 whole brain, skin and stomach. Notably, *Sox11 *sense transcripts are expressed more highly than antisense transcripts in the E15.5 whole brain and skin (23- and 4-fold, respectively).

**Figure 11:**
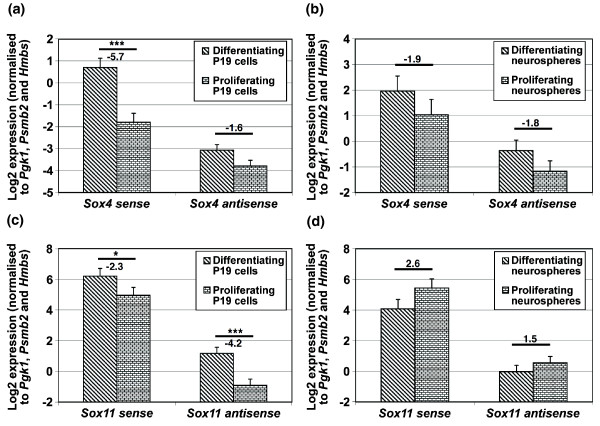
**Expression of *Sox4 *and *Sox11 *transcripts in neuropsheres and P19 cells**. **(a-d) **The figure shows strand-specific RT-qPCR screening of *Sox4 *(a, b) and *Sox11 *(c, d) sense and antisense transcripts expression in proliferating and differentiating P19 cells (a, c) and neuropsheres (b, d). N = 3 for P19 cells and N = 2 for neuropsheres. All data are presented as mean ± standard error of the mean. Fold change values (normalization to proliferating cells) are presented above the comparative bar and any values <1 are presented in the negative fold change format. * P < 0.05; ** P < 0.01; *** P < 0.001.

Since both Sox4 and Sox11 are implicated in neuronal differentiation and glial maturation processes [65,66], we examined both *Sox4 *and *Sox11 *sense and antisense transcript expression in proliferating and differentiating P19 (embryonal carcinoma cells) and in embryonic NSPCs grown as neurospheres. Both *Sox4 *sense and antisense transcripts are upregulated during P19 cell differentiation (approximately 5.7- and 1.6-fold upregulation, respectively; Figure 11a) and neurosphere differentiation (approximately 1.9- and 1.8-fold upregulation, respectively; Figure 11b). For *Sox11*, both sense and antisense transcripts are upregulated in the differentiating compared to the proliferating P19 cells by approximately 2.3- and 4.2-fold, respectively (Figure 11c). Both the *Sox11 *sense and antisense transcripts are, however, downregulated in the differentiating neurospheres (approximately 2.6- and 1.5-fold, respectively; Figure 11d).

## Discussion

In this study, SAGE was used to analyze global gene expression in the normal mouse cerebral cortex at various developmental stages. We report validated spatio-temporal regulation of genes involved in mouse cerebral cortex development from embryo to adulthood. The study highlights four main findings: association of DETs from different gene clusters with known functional processes or signaling pathways and disease-causative genes that are involved in cerebral corticogenesis; *Ankzf1 *and *Sox4 *sense transcripts are regionally expressed in the E15.5 cerebral cortex; multiple overlapping *Sox4 *and *Sox11 *sense and antisense transcripts are spatio-temporally regulated during cerebral corticogenesis; and *Sox4 *and *Sox11 *antisense transcripts are differentially regulated in both proliferating and differentiating embryonic-derived neurospheres and P19 cells.

We have shown that most tags generated in all libraries were singletons. The number of singletons could be reduced by increasing the number of tags sequenced. In mammalian cells, the number of additional unique transcripts identified approached zero when the number of SAGE tags sequenced reached approximately 600,000 [67]. Increasing the number of tags sequenced could improve the sensitivity of the technique to identify weakly expressed or novel transcripts, and the application of massively parallel signature sequencing [68] using a next-generation sequencer would be an ideal solution to accomplish this. In addition, one of the benefits of SAGE is that datasets generated from different groups or in public repositories such as SAGE Genie [69] and GEO [28] are readily comparable and, hence, can increase the tag count and sensitivity of the technique in discovering DETs between SAGE libraries. However, any meta-analyses involving various SAGE datasets are affected by experimental and biological variation; thus, a careful selection of matching libraries is crucial to limit systematic error or biases.

Our SAGE analysis robustly detected DETs with a low false positive rate (for example, <0.001% for comparison between left and right hemispheres of the adult cerebral cortex). Of all the identified DETs, approximately 8% were not mapped to either a single locus in the mouse genome or any unique annotation. This problem could be overcome by generating additional information from the 5' end of the transcript through alternative techniques such as PET sequencing [70], cap analysis gene expression (CAGE) [71] and 5' LongSAGE [72].

We have identified functional ontologies, molecular interactions and enriched canonical pathways that are distinct to the stage-specific gene clusters of validated DETs. The IPA network analysis generated connections between validated DETs across various developmental stages in relation to well-established proteins or molecules and neurological disorders. In the study, members of the Wnt/β-catenin signaling pathway were enriched in networks 1 and 2 (embryonic-specific gene clusters). In neural development, Wnt/β-catenin signaling plays an important role in regulating regional specification of the cortex along the rostro-caudal and dorso-ventral axes, and proliferation of cortical progenitors [73]. IPA highlighted three genes (*Sox4*, *Sox11 *and *Sfrp1*) associated with this pathway. In humans, the SFRP1 protein (secreted frizzled-related protein 1) stabilizes β-catenin and increases transcription from β-catenin-responsive promoters [74]. In β-catenin-deficient mouse mutants, expression of both *Sox4 *and *Sox11 *is downregulated [75,76]. Sox4 and Sox11 proteins play an important role in establishing neuronal properties, pan-neuronal gene expression and proper myelination of the central nervous system [65,66]. This suggests that the role of the Wnt/β-catenin signaling pathway in regulating neuronal development could be mediated, at least in part, by the Sox4 and Sox11 proteins.

The role of Wnt/β-catenin signaling in regulating DETs (*Btg1*, *Cdk4*, *Cdkn1c*, *Csrp2*, *Ezh2*, *Neurod1*, *Pcna*, and *Rps4x*) involved in cell cycle and proliferation remains unclear. Gain- and loss-of-function studies have established that Wnt/β-catenin signaling is essential to maintain the pool of precursors for proper development of the cerebral cortex [77,78]. To date, there is no direct evidence to show that *Ezh2*, *Pcna*, *Rps4x *and *Btg1 *are involved in cell cycle regulation during early embryonic neurogenesis. But their expression in the ventricular/subventricular zone of the E15.5 developing mouse cerebral cortex [33,35,36] suggests that they may be involved in regulating cell proliferation during neurogenesis. The role of these DETs and their association with the Wnt/β-catenin signaling pathway remains unclear and requires further experimentation.

Networks 3 and 4 from the adult gene cluster were associated with various canonical pathways, in particular, synaptic long term potentiation (LTP) and calcium signaling. LTP is a process of synapse enhancement, which is thought to underlie some forms of learning and memory [79]. This process depends on Ca^2+ ^and calmodulin, which are major components of the calcium signaling pathway. We identified four DETs, *Gria3*, *Itpr1*, *Ppp3ca *and *Camk2a*, in both canonical pathways. In particular, the Camk2a enzyme is highly expressed in the brain and regulates mainly glutamatergic synapses during LTP [79]. DET products such as Nrgn and Camk2n1 can directly or indirectly regulate Camk2a or Ca^2+^/calmodulin and subsequently alter the outcome of the LTP pathway [80,81]. Therefore, they may serve as important candidate genes in the future analysis of the synaptic LTP pathway involving neurodegenerative diseases that cause the loss of cognitive function and memory.

Networks 5 and 6 are enriched for genes in the amyloid processing signaling pathway. *App *and *Mapt *are associated with this pathway. Under normal circumstances, the App protein is required for proper migration of neuronal precursors into the cortical plate in early embryonic corticogenesis [82]. The Mapt protein, on the other hand, plays an important role in maintaining the architecture of the neuronal cytoskeleton and intracellular trafficking. Overexpression of App protein and hyperphosphorylation of the Mapt protein have been implicated in the pathologies of Alzheimer's disease [83,84]. Interestingly, *Ctsd*, *Atp7a*, *Clcn2 *and *Hprt1*, the genes responsible for other human neurological disorders such as neuronal ceroid lipofuscinosis (*Ctsd*), Menkes disease (*Atp7a*), epilepsy (*Clcn2*), and Lesch-Nyhan syndrome (*Hprt1*), are associated with *App *and *Mapt*. These candidate genes are also involved in cell morphogenesis, assembly and organization and could be linked to deterioration of neurons during the pathologic progression of these disorders.

Pathway analysis of DETs classified into the N, G and B groups showed DETs in the neuron (N and B groups) are associated with Huntington's disease and schizophrenia, which were not previously identified in networks 3 to 6. Our analysis showed that both disorders share three common DETs, namely *Rgs4*, *Ppp1r1b *and *Chgb*, whose expression is downregulated in humans with Huntington's disease or schizophrenia [85-88]. A proportion of patients with Huntington's disease also develop schizophrenia [89,90]. Taken together, downregulation of *Rgs4*, *Ppp1r1b *and *Chgb *expression in neurons may contribute to the common symptoms in these disorders. Our findings imply that many DETs (including *App*, *Hprt1 *and *Sncb*) associated with both Huntington's disease and schizophrenia are also involved in neuronal/cell death processes [91-93]. Other DETs in the N group, not previously implicated in neuronal cell death, may serve as novel potential candidates during pathologic development in these disorders.

Regionalized development of the cerebral cortex involves the differential regulation of cell cycle exit, early migration and attainment of positional identity in neuronal fated cells. To date, only few genes have been associated with regionalized development of the cerebral cortex [3,94]. In the regionalization analysis, we identified the highest number of DETs in the comparison of the RL and CM libraries, which signifies that these two regions of the cerebral cortex are the most different. This finding supports the notion that the cerebral cortex is developed in a latero-medial axis followed by a rostro-caudal axis [7,8]. At E15.5, both *Actb *and *Tmsb4x *were expressed greater in the rostral cerebral cortex than in the caudal region. Both Actb and Tmsb4x proteins are involved in the actin cytoskeleton-signaling pathway [95]. In particular, the Tmsb4x protein has been shown to promote cardiomyocyte migration [96] and axonal tract growth in zebrafish [97]. Therefore, co-expression of *Actb *and *Tmsb4x *in the E15.5 mouse cortical plate suggests that they may have a synergistic role in early cortical cell development. Conversely, *Blcap *and *Ankzf1 *were expressed more highly in the caudal than in the rostral region of the E15.5 cerebral cortex. To date, the function of both *Blcap *and *Ankzf1 *in the cerebral cortex remains uncharacterized. This study provides the first comprehensive expression profile of *Ankzf1 *and suggests it could be an important transcription factor in cerebral corticogenesis.

At E15.5, *Sox4 *sense transcripts were expressed in a high-rostral and high-caudal manner with lesser expression within the intermediate region. By E17.5, *Sox4 *expression becomes obvious at the rostral cortical plate, which is similar to *Rorb*. But, we did not find that the regionalized expression of *Sox4 *sense transcripts resembles that of the restricted *Rorb *expression at E15.5 or in the postnatal brain [1,62]. This finding could be caused by the combined expression profiles of different *Sox4 *sense transcripts that are present across the rostro-caudal axis of the cortical plate. The regionalized expression of *Sox4 *sense transcripts occurs only between E15.5 and E17.5. Because the thalamic axon innervates the cortical plate after E17.5 [98], the regionalization of *Sox4 *sense transcripts in early cortical development could be an outcome of an intrinsic instead of an extrinsic mechanism that regulates early patterning of the cerebral cortex.

Genomic clustering of DETs identified the differentially regulated *Sox4 *and *Sox11 *gene loci. These genomic clusters imply that there are multiple overlapping sense and antisense transcripts surrounding the same gene locus that are co-transcribed simultaneously during cerebral cortex development. Both *Sox4 *and *Sox11 *are single exon genes and these transcript variants are therefore likely to be generated due to alternative polyadenylation. The 3' UTRs of both *Sox4 *and *Sox11 *have tandem terminal polyadenylation signals on both sense and antisense strands (data not shown), which supports the occurrence of multiple transcript forms or SAGE tags. Multiple mRNA forms with different 3' UTRs can lead to cell-specific regulation, different nuclear or cytoplasmic mRNA stability and translation rates [99,100]. The 3' UTR of *Sox4 *and *Sox11 *may contain AU-rich elements that play an important role in determining mRNA stability through deadenylation, decapping or 3' → 5' decay [101]. Besides, different 3' UTR lengths may be targeted by different miRNAs, thus interfering with the translation process. Both *Sox4 *and *Sox11 *transcripts may be targeted by various miRNAs at different predicted positions across the 3' UTR (Tables S7 and S9 and Figures S15 and S18 in Additional data file 1). Therefore, 3' UTR lengths of *Sox4 *and *Sox11 *may be an important feature in the regulation of their protein expression during cerebral corticogenesis.

In the study, NATs were found at both the *Sox4 *and *Sox11 *gene loci overlapping the sense transcripts. Overlapping NATs may function as templates for the generation of pre-miRNA and mature miRNA with exceptional high sequence conservation that complement the overlapping sense protein-coding transcripts [102]. To date, no mature or pre-miRNAs have been predicted on the *Sox4 *and *Sox11 *sense and antisense strands (data not shown) or have been reported in miRBase [103]. In addition, NATs can self-complement to form double stranded RNA or pair with sense transcripts and function as templates for the generation of endogenous small interfering RNAs, which could subsequently interfere with translation or transcription of multiple protein-coding transcripts [104]. Because both Sox4 and Sox11 proteins are highly expressed in the cerebral cortex, the overlapping NATs do not seem to be involved in the regulation of Sox4 and Sox11 through miRNA- or small interfering RNA-mediated translation repression mechanisms, but rather through antisense-regulated sense transcription within the nucleus.

Our ISH analysis showed complementary cellular expression profiles of *Sox4 *sense and antisense transcripts at the piriform cortex, olfactory bulb and dentate gyrus. This finding implies that *Sox4 *antisense transcripts may be essential in intracellular and interlocus negative feedback loop regulation of the *Sox4 *sense transcripts. Similar expression profiles of *Sox4 *sense and antisense transcripts in multiple mouse organs and brain regions, however, suggest that these transcripts may be co-expressed. This observation is also supported by the temporal co-expression of *Sox4 *sense and antisense transcripts in the cortical plate or layers I to III of the cerebral cortex. Taken together, the sense and antisense transcripts of *Sox4 *are co-expressed in some cells and expressed complementarily in other cells, suggesting crucial cell-type-specific regulation.

Sox4 and Sox11 have been shown to have redundant roles during mouse development [105], and Sox11 may play a compensatory role in the absence of Sox4 during brain development [106]. We demonstrated that *Sox4 *and *Sox11 *sense and antisense transcripts have a similar expression in the brain, but not in other organs, suggesting a compensatory role for Sox11 only in the brain. *Sox11 *antisense transcripts were expressed in the brain, skin and stomach only, suggesting organ-specific regulation.

Our data show upregulation of *Sox4 *and *Sox11 *sense transcripts in differentiating P19 cells, consistent with the findings of others [107,108], and demonstrate upregulation of antisense transcripts as well. We also find both *Sox4 *and *Sox11 *sense transcripts expressed in the NSPCs cultured as neurospheres, which is in agreement with Dy *et al*. [109]. Furthermore, we identify upregulation of both *Sox4 *sense and antisense transcripts but downregulation of *Sox11 *sense transcripts in differentiating neurospheres. Taken together, our findings show that there are potentially common and distinct roles for *Sox4 *and *Sox11 *sense and antisense transcripts during neuronal and non-neuronal cell proliferation and differentiation. The underlying regulatory mechanism of these transcripts, particularly the antisense ones, remains unknown and requires further investigation.

## Conclusions

This study provides avenues for future research focus in understanding the fundamental processes and development of neurological disorders related to the cerebral cortex. We confirm the regionalized expression of new candidate genes in the E15.5 cerebral cortex as well as differential regulation of multiple overlapping sense and novel antisense transcripts within *Sox4 *and *Sox11 *gene loci during cerebral corticogenesis. We also report for the first time the spatio-temporal regulation of *Sox4 *antisense transcripts in the brain as well as differential regulation of novel *Sox4 *and *Sox11 *antisense transcripts in various mouse organs and in proliferating and differentiating NSPCs and P19 cells. The finding provides an insight for future investigations into the role of antisense transcripts during cerebral corticogenesis and neuronal differentiation.

## Materials and methods

### Serial analysis of gene expression

#### Handling of animals and dissection of the cerebral cortex

All experiments that involved animal breeding and handling were performed according to protocols approved by the Melbourne Health Animal Ethics Committee (Project numbers 2001.045 and 2004.041). All animals involved in the study were C57BL/6 mice unless specified otherwise. All mice were kept under conditions of a 12-h light/12-h dark cycle with unlimited access to food and water. All mice were culled by cervical dislocation prior to dissection. Cortical tissue was procured in the following fashion. For adult samples, after removal of the meninges, coronal cuts were used to excise the olfactory bulb from the rostral region, and the superior colliculus from the caudal region. A sagittal cut to separate the two cortical hemispheres was performed. The cortical pallium was dissected from the subpallial striatum and the septum. The neocortex was then dissected away from the cingulate cortex and the entorhinal cortex. For embryonic samples, the cortical tissue was dissected free from the underlying ganglionic eminences at the pallial-subpallial border. An orthogonal cut was made to remove the presumptive striatum and the overlying piriform cortex. On the medial aspect, the medial limbic cortex was included for analysis, but the adjacent hippocampal primordium, including the cortical hem, was excluded. For the E15.5 cerebral cortex, the resulting hemispheres containing cortical tissue only were placed on the bottom of the Petri dish and, using a fine scalpel, divided into four equal quadrants per hemisphere, namely RL, RM, CL and CM. Rostral and caudal quadrants from both hemispheres were pooled for SAGE library construction but separately tracked for RT-qPCR analysis. Procurement of other adult brain tissues and related mouse organs for *Sox4 *and *Sox11 *antisense transcript screening was carried out according to the standard mouse necropsy protocol accessible at the National Institute of Allergy and Infectious Diseases (NIAID) website [110].

#### SAGE libraries and analysis of tags

Ten SAGE libraries were constructed from the cerebral cortex of E15.5, E17.5 and 4- to 6-month-old (Ad) mice according to either one of the two methods described previously [25,26], using I-SAGE™ or I-SAGE™ Long Kits (Invitrogen, Mulgrave, Victoria, Australia). Additional libraries from E15.5 and P1.5 of the cerebral cortex described previously [27] were also included in the analysis. These libraries contain a total of 26,436 traces. SAGE tags were preprocessed - that is, TAGs were extracted and corrected for sequencing errors, and artifacts like SAGE linkers, ribosomal RNA and duplicated ditags were removed using the 'sagenhaft' package, which is available from the Bioconductor website [111,112]. To compare libraries that contain long tags with those that contain short tags, all short tags were mapped to the existing long tags from the other libraries. A table for all libraries containing the unique long or short tags was generated and redundant tags were removed. Only tags with a total count >2 (across all libraries) were considered for subsequent comparisons. Each unique tag was mapped to the mouse genome using ESTgraph, which employs ESTs and their genomic position information. ESTgraph was created by Tim Beissbarth (unpublished) [113]. Identity was assigned to these tags and they were further grouped into the following categories: matching to a gene, a genomic sequence, or an EST, or ambiguous matches or no alignment at all. All annotations were based on the latest mouse assembly (mm9 released in July 2007) accessible from the UCSC Genome Bioinformatics website [63].

#### Identification of differentially expressed tags

Library comparisons were performed using two methods. Fisher's exact test was used to compare two individual SAGE libraries. In the analysis, multiple testing correction [114] was carried out to control for false-discovery rate and adjusted *P*-value cutoffs (Q-values) were used to select DETs. In cases where several libraries were combined to focus on a specific biological comparison (for example, different stages of development), a Bayesian model, as described previously [115], was used to integrate multiple libraries in pairwise comparisons involving biological replicates of libraries. The model accounts for within-class variability by means of mixture distributions. The resulting E-values were used to select DETs. A table of all relevant comparisons, the comparison method and Q- or E-value cutoffs is provided in Table S1 in Additional data file 1.

#### Hierarchical clustering of SAGE tags

To identify co-regulated genes, the clustering of DETs was performed based on the log2 of normalized counts. Each library was normalized to 100,000 tags per library to account for size differences. A pseudocount of 0.5 was added before taking the log2 of the normalized tag counts. The tag-wise mean was subtracted from the log2 tag intensities before computing the Euclidean distance of the individual tag profiles. Hierarchical clustering was performed on the tags using the 'hclust' function and complete linkage, which was implemented using the statistical computing environment of R [116].

#### Genomic clustering of SAGE tags

To assess whether there was any genomic clustering of tags, a method previously described [117] was adopted. In brief, first gene lists (based on all DETs in both pairwise and multiple library comparisons as well as gene lists from the hierarchical clustering analysis) were selected. The genomic clustering of either of these selections was compared to the total unique tag list (all 25,165 unique tags). The tags were mapped to the mouse genome. The number of selected tags in ten consecutive tag positions for each window of the chromosome was calculated. One thousand permutations were used to compute the null distribution of maximum tag counts per window. The method was implemented using the statistical computing environment of R [116].

### Functional classification and characterization of DETs

#### Gene Ontology enrichment analysis

The DET lists generated from various comparisons were subjected to systematic functional annotation using the standardized Gene Ontology term analysis tools at the DAVID [30]. Functional clustering was performed using high stringency with a kappa similarity threshold of 0.85 and a minimum term overlap of 3. Classification was carried out using a multiple linkage threshold of 0.5 with both numbers of initial and final group members set to 3. A term was considered statistically significant when the computed *P*-value was < 0.05. All queries were performed in September 2009.

#### Molecular interactions and pathway analysis

Identification of molecular network interactions and pathway analysis of validated DETs or co-regulated genes was completed using the IPA [118] tools from Ingenuity Systems^® ^(Redwood City, California, USA). Accession numbers for all genes with their corresponding fold changes or normalized counts were imported into the IPA software. No focus genes were set at the beginning of the analysis. To start building networks, the application queries the list of input genes and all other gene objects stored in the Ingenuity knowledge base. Networks with a maximum of 30 genes or proteins were constructed, and scores were computed based on the likelihood of the genes being connected together due to random chance. A score of 2 indicates that there is a 1/100 chance that these genes are connected in a network due to random chance. Therefore, any networks with a score of 2 or above are considered statistically significant (with >99% confidence). The most significant novel networks and their interactions with existing canonical pathways were investigated further.

### Quantitative RT-PCR

#### Total RNA isolation and first strand cDNA synthesis

RT-qPCR was carried out to validate all selected candidate or co-regulated DETs. Biological triplicates from E15.5, E17.5, P1.5 and adult (5 to 6 months old) cerebral cortices were used. Genomic-free total RNA from independent mice was extracted using the RNeasy^® ^Lipid Tissue Midi Kit (Qiagen, Doncaster, Victoria, Australia) according to the manufacturer's protocol (Section G in Additional data file 1). First strand cDNA was synthesized from high quality total RNA [119] using random hexamers (unless specified otherwise) and the SuperScript™ III Reverse Transcriptase Kit (Invitrogen, Mulgrave, Victoria, Australia), according to the manufacturer's protocol, under an RNAse-free environment.

#### Primer design and RT-qPCR

All primers were designed using ProbeFinder 2.35 at the Assay Design Center for Universal ProbeLibrary Assay provided by Roche Applied Science [120]. All RT-qPCR reactions were prepared in 10 μl volumes in a 384-well plate format consisting of LC480 Master Probe Mix (Roche Diagnostics, Castle Hill, New South Wales, Australia), Universal ProbeLibrary (Roche Diagnostics, Castle Hill, New South Wales, Australia) and forward and reverse primers (GeneWorks, Hindmarsh, South Australia, Australia or Bioneer Corporation, Daedeok-gu, Daejeon, Korea) according to the manufacturers' protocols. RT-qPCRs were performed using the LightCycler^® ^480 System (Roche Diagnostics, Castle Hill, New South Wales, Australia) according to the manufacturer's protocol (Section G in Additional data file 1). A full list of primers and probes used in this study is included in Additional data file 8.

#### Relative quantification using a standard curve method

The crossing point (Cp) from each signal was calculated based on the Second Derivative Maximum method [121]. A set of serially diluted cDNAs was used to construct a four-data point standard curve for every PCR system in each run. A total of three reference genes (from *Hprt1*, *Psmb2*, phosphoglycerate kinase 1 gene (*Pgk1*) *or Hmbs*) were used as endogenous controls. An estimated starting amount of each target gene was calculated and intra-samples multiple reference genes normalization was performed (Section G in Additional data file 1). A linear model was fitted to the time course of expression values for each gene. Genes differentially expressed between the various stages of development or regions were selected using empirical Bayesian moderated t-statistics, which borrow information between genes [122]. Standard errors for the mean expression at various developmental stages were obtained from the linear model. For each comparison, *P*-values were adjusted using the Benjamini and Hochberg [114] method to control the false discovery rate. See Section H in Additional data file 1 for the R code used to execute the analysis.

### Validation of sense-antisense and multiple overlapping transcripts in genomic clusters

#### Strand specific RT-PCR

All RNA was prepared as described above. Total RNA from all developmental stages (N = 3 per developmental stage) was equally pooled prior to cDNA synthesis. Four first strand cDNA synthesis reactions were prepared for each cluster as follows: with a primer complementary to the sense strand only; with a primer complementary to the antisense strand only; with oligo-d [T]_15 _as a positive control; and without any primers as a negative control. In all four reactions, both primers were added in subsequent PCRs (Section G in Additional data file 1). PCR amplifications were carried out using FastStart PCR High Fidelity System (Roche Diagnostics, Castle Hill, New South Wales, Australia) according to the manufacturer's protocol. More than one primer set was used in the sense-antisense strand specific RT-PCR (Additional data file 8).

#### RACE

First strand cDNA synthesis was carried out using pooled total RNA extracted from three biological replicates of rostral and caudal E15.5 and whole E15.5, E17.5, P1.5 and adult (5 to 6 months old) cerebral cortices. Oligo-d [T]_15 _with an adaptor sequence (5'-TACGACGTCTGCTAGGACTG-3') was used to prime the first strand cDNA synthesis. Second strand synthesis or PCR was then carried out using a strand-specific primer and the adaptor primers (Additional data file 8). All specific primers were designed to be complementary to the SAGE tags or their upstream sequences. PCR amplifications (Section G in Additional data file 1) were carried out using FastStart PCR High Fidelity System (Roche Diagnostics, Castle Hill, New South Wales, Australia) according to the manufacturer's protocol.

#### Southern blotting analysis

Amplified 3' RACE products were transferred to Hybond N^+TM ^(GE Healthcare, Rydalmere, New South Wales, Australia) nylon membrane using the neutral transfer method. Prehybridization and hybridization steps were performed in Rapid-Hyb buffer (GE Healthcare, Rydalmere, New South Wales, Australia) according to the manufacturer's protocol. All oligonucleotides were designed to be complementary to sequence between the specific primer-priming site and the tag of interest. Synthetic oligonucleotides were 5' end-labeled using T4 polynucleotide kinase (Promega, Alexandria, New South Wales, Australia) and [γ-^32^P]ATP (GE Healthcare, Rydalmere, New South Wales, Australia) with modifications to the manufacturer's protocol. After the hybridization step, the membrane was washed with 5× sodium chloride sodium citrate solution (with 0.1% v/v sodium dodecyl sulphate (SDS)) and 1× sodium chloride sodium citrate solution (with 0.1% w/v SDS) (Section G in Additional data file 1). See Additional data file 8 for primer sequences and oligonucleotides used for detection.

#### Northern blotting analysis

Independent preparations of total RNA from the cerebral cortex of seven mice at E15.5 and E17.5, and three adult mice were equally pooled to a final concentration of 20 μg per developmental stage. These pooled total RNAs were electrophoresed overnight and capillary transferred onto Hybond N^+TM ^(GE Healthcare, Rydalmere, New South Wales, Australia) nylon membrane. Double-stranded DNA probes were radioactive-labeled using the Amersham Megaprime DNA Labeling System (GE Healthcare, Rydalmere, New South Wales, Australia) and [β-^32^P]CTP, according to the manufacturer's protocol. Hybridization was carried out overnight (approximately 18 h) at 65°C in aqueous buffer (7% w/v SDS with 0.5 M phosphate). After hybridization, blots were washed using 1% w/v SDS at 65°C for 5 to 6 times until the background signal was low.

#### *In situ *RNA hybridization

ISH was carried out using paraffin sections (5 μm) of embryonic, postnatal and adult brains (E11.5, E13.5, E15.5, E17.5, P1.5 and P150) and a related [^35^S]UTP-labeled complementary RNA probe (Additional data file 8) as described previously (Section G in Additional data file 1) [123].

### Screening of *Sox4 *and *Sox11 *sense and antisense transcript expression in the adult mouse brain, organs and both the proliferating and differentiating P19 cells and neurospheres

#### Strand-specific RT-qPCR

Total RNA was extracted from harvested organs using the TRIzol^®^'s reagent (Invitrogen, Mulgrave, Victoria, Australia) according to the manufacturer's protocol. To avoid genomic DNA contamination, all isolated total RNA was treated with the recombinant DNAse I enzyme provided by the DNA-*free*™ kit (Applied Biosystems, Scoresby, Victoria, Australia) according to the manufacturer's protocol. First strand cDNA synthesis was carried out using strand-specific primers followed by qPCR analysis as described above.

#### Embryonic neural stem cells grown as neurospheres

Mouse used for generation of neurospheres had a mixed genetic background including MF1, 129SvEv, C57BL/6 and CBA. Cerebral cortices from E14 embryos were dissected out into calcium-magnesium-free phosphate-buffered saline. The tissue was mechanically dissociated and centrifuged. The cells were plated in complete neuroculture medium (Section G in Additional data file 1) for 4 days followed by induction of neuronal differentiation. These cells were then plated on poly-D-lysine (catalogue number P6407, Sigma Aldrich, Castle Hill, New South Wales, Australia) and laminin (catalogue number 23017-015, Invitrogen, Mulgrave, Victoria, Australia) coated culture dishes in neuroculture medium with the presence of 2% (v/v) fetal bovine serum but not epidermal growth factor and basic fibroblast growth factor. The differentiation was allowed to proceed for 5 days. Total RNA was extracted from both proliferating and differentiating cells using TRIzol^® ^reagent as described above.

#### P19 embryonal carcinoma cells

P19 mouse embryonal carcinoma cells were cultured and differentiated into neurons as described previously [124]. Briefly, P19 cell cultures were maintained in P19GM complete medium (Section G in Additional data file 1). For induction of neuronal differentiation, 1 × 10^6 ^P19 cells were cultured in suspension form using bacteriological Petri dishes. The P19GM medium with additional supplementation of 5 × 10^-7^M all-trans retinoic acid (catalogue number R-2625; Sigma Aldrich, Castle Hill, New South Wales, Australia) was used for the induction. After 4 days, P19 cells formed embryoid body stages. Embryoid bodies were collected from suspension cultures and re-plated in adherent culture flasks in the P19GM medium with only 5% (v/v) fetal bovine serum and without retinoic acid supplementation. The cells were allowed to differentiate for 5 days. Total RNA was extracted from both proliferating and differentiating cells using TRIzol^® ^reagent as described in above.

## Abbreviations

CL: caudo-lateral; CM: caudo-medial; DAVID: Database for Annotation, Visualization and Integrated Discovery; DET: differentially expressed transcript/tag; E: embryonic day; EST: expressed sequence tag; GEO: Gene Expression Omnibus; IPA: Ingenuity Pathway Analysis; ISH: *in situ *hybridization; LTP: long term potentiation; miRNA: microRNA; NAT: natural antisense transcript; NSPC: neural stem/progenitor cell; OMIM: Online Mendelian Inheritance in Man; P: postnatal day; PET: paired-end diTag; RACE: rapid amplification of cDNA ends; RL: rostro-lateral; RM: rostro-medial; RT-qPCR: quantitative RT-PCR; SAGE: serial analysis of gene expression; SDS: sodium dodecyl sulphate; UCSC: University of California Santa Cruz; UTR: untranslated region.

## Authors' contributions

KHL performed all the SAGE validation experiments. CAH, PZC and SST procured the mouse cerebral cortex and constructed the SAGE libraries. KHL, TB, LH and GKS designed, performed and supervised the SAGE, RT-qPCR and IPA analyses. KHL and TT performed all the ISH studies. KHL, KB, PSC, CNH and PQT carried out the expression studies on *Sox4 *and *Sox11 *transcripts. KHL, CAH and CNH drafted the manuscript. CAH, GKS, TT and HSS conceived of the study, and participated in its design and coordination. All authors read and approved the final manuscript.

## Additional data files

The following additional data are available with the online version of this paper: analysis of SAGE, DETs, IPA, *Sox4 *and *Sox11 *genomic cluster analysis, and R script for implementing empirical Bayesian moderated *t*-test on multiple groups (Additional data file 1); SAGE tag information for 561 DETs (Additional data file 2); functional annotations clustering analysis using DAVID (Additional data file 3); RT-qPCR validation of DETs based on multiple comparisons between two developmental stages (E versus Ad, PN1.5 versus Ad and E15.5 versus PN1.5) (Additional data file 4); RT-qPCR validation of gene clusters based on hierarchical clustering analysis (Additional data file 5); RT-qPCR validation of DETs based on the rostral versus caudal E15.5 cerebral cortex comparison (Additional data file 6); statistically significant over-represented genomic loci based on genomic clustering of tags (Additional data file 7); list of primers, probes, clones and assays designed for RT-qPCR, RACE, Southern, Northern and ISH analysis (Additional data file 8).

## Supplementary Material

Additional data file 1 Analysis of SAGE, DETs, IPA, *Sox4 *and *Sox11 *genomic cluster analysis, and R script for implementing empirical Bayesian moderated *t*-test on multiple groups. Click here for file

Additional data file 2 SAGE tag information for 561 DETs. Click here for file

Additional data file 3 Functional annotations clustering analysis using DAVID. Click here for file

Additional data file 4 RT-qPCR validation of DETs based on multiple comparisons between two developmental stages (E versus Ad, PN1.5 versus Ad and E15.5 versus PN1.5). Click here for file

Additional data file 5 RT-qPCR validation of gene clusters based on hierarchical clustering analysis. Click here for file

Additional data file 6 RT-qPCR validation of DETs based on the rostral versus caudal E15.5 cerebral cortex comparison. Click here for file

Additional data file 7 Statistically significant over-represented genomic loci based on genomic clustering of tags. Click here for file

Additional data file 8 Primers, probes, clones and assays designed for RT-qPCR, RACE, Southern, Northern and ISH analysis. Click here for file
