# Supplementary material for: Molecular networks involved in mouse cerebral corticogenesis and spatio-temporal regulation of Sox4 and Sox11 novel antisense transcripts revealed by transcriptome profiling
Source: Genome Biol. 2009 Oct 2;10(10):R104. doi: 10.1186/gb-2009-10-10-r104 (PMC2784319; doi:10.1186/gb-2009-10-10-r104)
Supplement: Additional data file 1 — Analysis of SAGE, DETs, IPA, Sox4 and Sox11 genomic cluster analysis, and R script for implementing empirical Bayesian moderated t-test on multiple groups. [file gb-2009-10-10-r104-S1.DOC]

***Additional data file 1*:**

***Analysis of SAGE, DETs, GO terms, Ingenuity Pathway Analysis, Sox4 and Sox11 genomic cluster analysis, additional description for Materials & Methods section and R script for implementing empirical Bayesian moderated T-test on multiple groups.***

**SECTION A: SERIAL ANALYSIS OF GENE EXPRESSION**


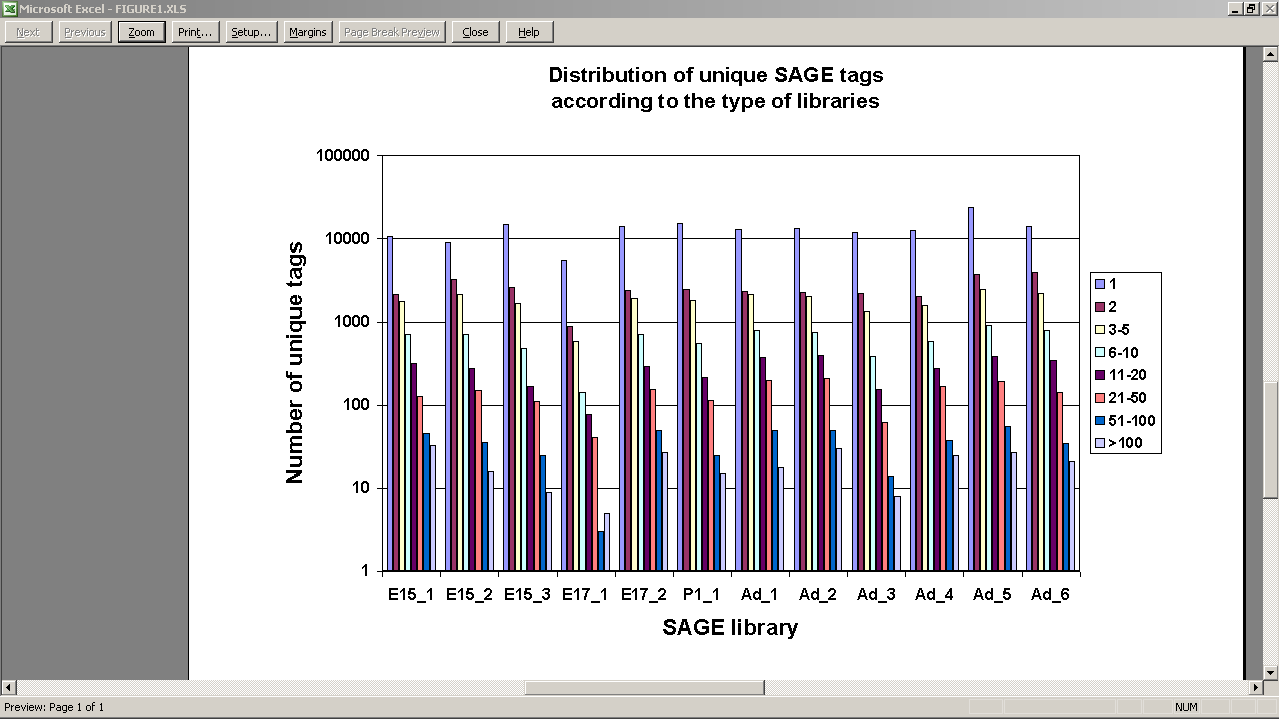


**Figure S1 Distribution of unique SAGE tags according to library type. The tag count represented by each bar is shown in the box on the right.**


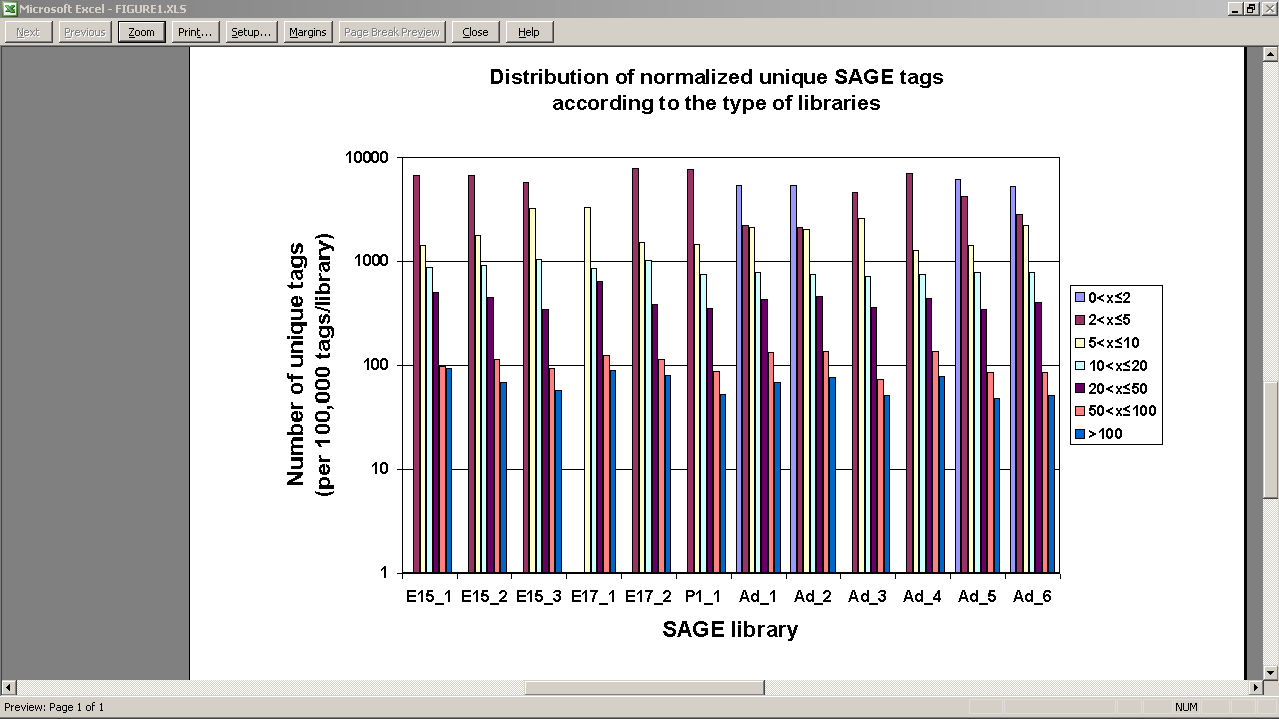


# Figure S2 Distribution of normalized unique SAGE tags (per 100,000 tags/library) according to library type. The tag count represented by each bar is shown in the box on the right.

#
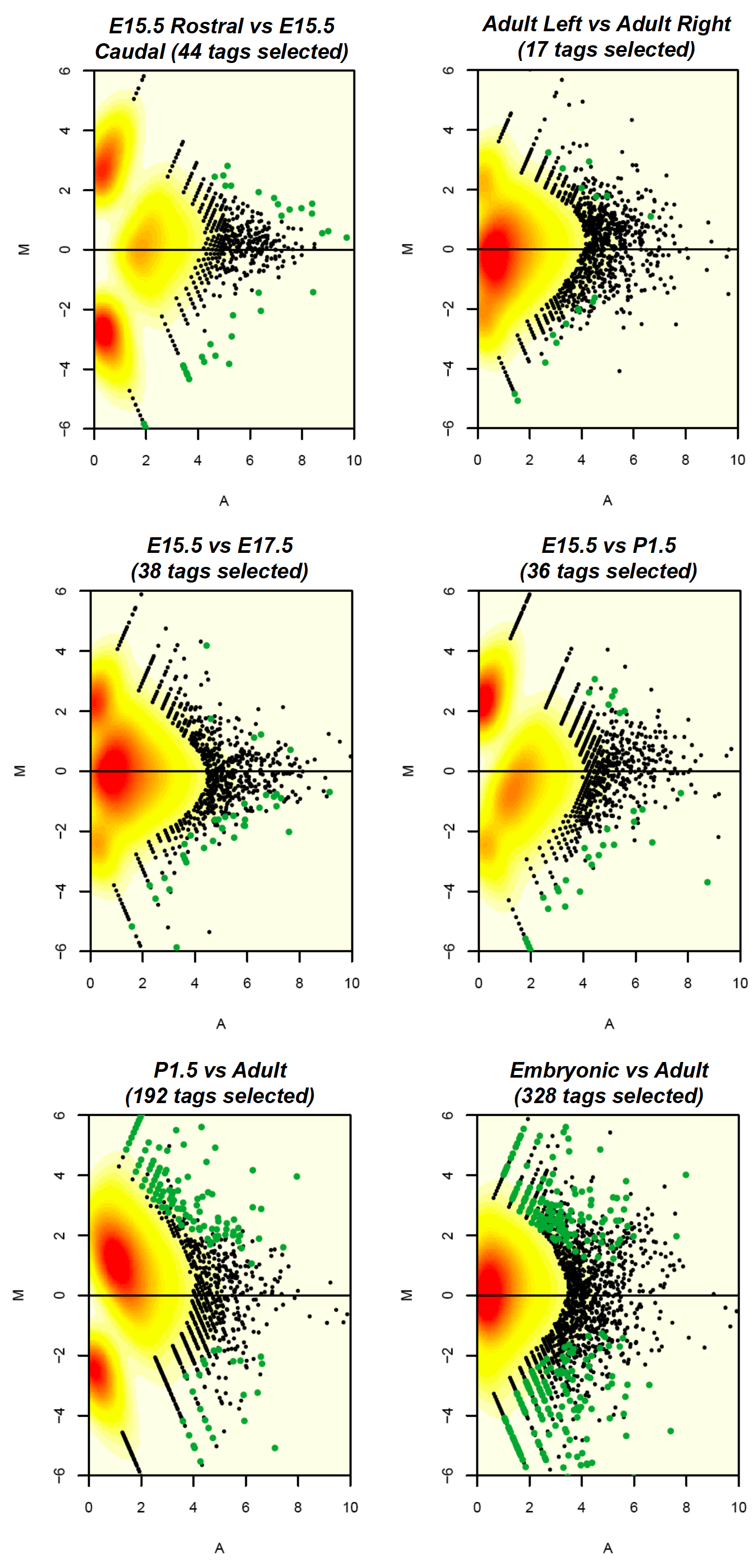


Figure S3 MA plots of various comparisons. The Y-axis represents the M values, which is the ratio (log2(P/Q)) whereas the X-axis represents the A values, which is the mean ratio (1/2*log2(P*Q)). P and Q represent the tag counts from the two libraries in the comparison. Tags are represented by black dots and those highlighted in green are significantly differentially expressed (see Table S1 for cutoff values). In dense regions the dots are
replaced by a heatmap, where the colour (from yellow to red) represents the density of dots. E denotes ‘embryonic days/stages’; P denotes ‘postnatal days’.

**Table S1: Statistical methods and cutoff values used in various comparison of two developmental stages**

| **No.** | **Comparison** | **Statistical method** | **Q or E value cutoff*** | **Number of DETs** |
| --- | --- | --- | --- | --- |
| 1. | R vs C (E15.5) | Fisher’s exact test | 0.1 | 44 |
| 2. | L vs Ri (Adult) | Vencio et al, 2004 | 0.001 | 17 |
| 3. | E15.5 vs E17.5 | Vencio et al, 2004 | 0.001 | 38 |
| 4. | E15.5 vs P1.5 | Vencio et al, 2004 | 0.001 | 36 |
| 5. | P1.5 vs Ad | Vencio et al, 2004 | 0.001 | 192 |
| 6. | E vs Ad | Vencio et al, 2004 | 0.001 | 328 |

* Q indicates p-value cutoffs based on Fisher’s exact test with multiple testing correction to control for the false-discovery rate [114]. E-value cutoffs were used in the Bayesian model as described previously [115]. R denotes ‘rostral region’; C denotes ‘caudal region’; L denotes ‘ adult left hemisphere’; Ri denotes ‘adult right hemisphere’; E denotes ‘embryonic days/stages’; P denotes ‘postnatal days’; Ad denotes ‘adult stage’

**SECTION B: SUMMARY OF RT-qPCR VALIDATION ANALYSIS**

RT-qPCR confirmed ~50% of SAGE expression profiles (*p*<0.05; Table S2). The majority of fold changes confirmed by RT-qPCR were lower than SAGE. These results indicate that at medium to high expression levels, SAGE provides a reliable estimation of differential gene expression. This is likely to be due to the normalization of libraries of various sizes to 100,000 tags, which could overcompensate the magnitude of differences between transcripts. In addition, technical differences between SAGE and RT-qPCR may also contribute to the fold change differences.

**Table S2: RT-qPCR validation of SAGE tags in various comparisons**

| Items | **E15.5**  **R vs C** | **Adult**  **L vs Ri** | **E15.5 vs. E17.5** | **E15.5 vs. P1.5** | **P1.5 vs**  **Ad** | **E**  **vs**  **Ad** | Total^b^ |
| --- | --- | --- | --- | --- | --- | --- | --- |
| No. of candidate DETs | 27 | 14 | 3 | 3 | 29 | 66 | 142 |
| **No. of DETs with gene ID** | 24 [8,3] | 10 [4,0] | 3 [0,3] | 3 [2,1] | 24 [21,16] | 54 [44,42] | 118 [79,65] |
| **No. of DETs with EST ID** | 1 [0,0] | 2 [0,0] | 0 [0,0] | 0 | 5 [4,3] | 9 [8,7] | 17 [12,10] |
| **No. of ambiguous DETs with ID** | 2 [1,0] | 2 [1,0] | 0 [0,0] | 0 | 0 [0,0] | 3 [2,2] | 7 [2,2] |
| **Failed RT-qPCR** | 4/27 | 1/14 | 0/3 | 0/3 | 0/31 | 9/66 | 14/144 |
| **Validation rate according to trend only** | 9/27 | 5/14 | 3/3 | 3/3 | 25/31 | 54/66 | 99/144 |
| **Validation rate according to trend with *p*<0.05^a^** | 3/27 | 0/14 | 0/3 | 2/3 | 19/31 | 51/66 | 75/144 |

[x,y] denotes x number of genes which were validated according to trend and y number of x genes were significantly different with a *p*<0.05 cutoff.

## ^a^ based on empirical Bayesian moderated T-test with Benjamini-Hochberg correction for FDR.

^b^ the final total values are higher than the one reported in the text because the calculation includes the same DETs that were differentially expressed in comparisons of various stages. The total number of unique DETs was 136 and the total number of unique DETs validated according to trend with an adjusted *p*<0.05 was 70.

Note: R - ‘rostral region’; C - ‘caudal region’; L - ‘ adult left hemisphere’; Ri - ‘adult right hemisphere’; E - ‘embryonic days/stages’; P - ‘postnatal days’; Ad - ‘adult stage’.

**SECTION C: INGENUITY PATHWAY ANALYSIS OF VALIDATED DETs**

**Table S3: Novel molecular networks, related biological functions/diseases and canonical pathways based on Ingenuity Pathway Analysis**

| **Gene clusters** | **Novel molecular networks** | | | | **Canonical pathway^e^** | ***p* value** |
| --- | --- | --- | --- | --- | --- | --- |
|  | **Focus gene^a^** | **Other associated nodes^b^** | **Biological functions and/or diseases^c^** | **Score^d^** |  |  |
| *Embryonic specific gene clusters (1, 5 and 6)* | ALS2CR2, BTG1, H2AFY, **MARCKS**, **NEUROD1**, ROBO1, RPS4X, SFRP1, SOX4, SOX11, WSB1 | BIK, CDX1, COTL1, CTNNB1, FGF4, FZD6, GHRHR, HOXB9, HUS1, IL15, MAPK8, MAPK12, PKMYT1, retinoic acid, RUVBL2, SDCBP, Sox, SUFU, T, TNF, TPT1, WNT2, WNT4, ZEB2 | Cellular Development, Organismal Development, Cellular Growth and Proliferation | 26 | Wnt/β-catenin signaling  P53 signaling  Tight junction signaling | 1.83E-03  8.13E-03  2.23E-02 |
|  | ACTB, 5031439G07Rik, CDK4, CDKN1C, CSRP2, DCX, EZH2, **MARCKS**, **NEUROD1**, PCNA | CCNDBP1, CCNO, CCT8, CDK4/6, COTL1, Cyclin A, Cyclin D, DAB2IP, DMTF1, ELP5, EMD, F Actin, FGF17, Histone h3, KIAA0101, LIG1, NFYB, NKX6-1, PAX7, PDGF BB, POLE, POU2F3, Rb, T, TSPYL2 | Cell Cycle, DNA Replication, Recombination, and Repair, Endocrine System Development and Function | 23 |  |  |
| *Adult specific gene clusters (4, 8 and 10)* | CAMK2A, EGR1, ITPR1, **MBP**, NRGN, PLP1, PPAP2B, PPP1R1B, PPP3CA, RGS4, SEPT4, SIRPA, SNCB | AHCYL1, Akt, Alkaline Phosphatase, BMP2K (includes EG:55589), CABP1, Calmodulin, CAMK2N2, CCKAR, Cytokine Receptor, Jnk, MAD2L2, MAP4K2, Mapk, NPLOC4, P38 MAPK, PPP3R2, STK39, TAOK2 (includes EG:9344), TRHR, Ubiquitin, UFD1L | Nervous System Development and Function, Neurological Disease, Cell Morphology | 33 | Synaptic Long Term Potentiation  Calcium Signaling  B Cell Receptor Signaling  cAMP-mediated Signaling  GM-CSF Signaling  Amyotrophic Lateral Sclerosis Signaling  Synaptic Long Term Depression  G-Protein Coupled Receptor Signaling  Xenobiotic Metabolism Signaling | 1.54E-05  9.60E-05  9.89E-04  1.30E-03  3.20E-03  7.68E-03  1.78E-02  3.03E-02  4.60E-02 |
|  | CRYAB, GRIA3, GSTM5 (GSTM1), **MBP**, NPTXR, TSPAN7 (TM4SF2) | ACTN1, AGTR2, CHRM2, DYRK1A, EDN3, ELK3, EPOR, FGF2, Fgfr, FOSL1, HOXD3, HSPB1, HSPB2, MAPK14, MYC, NAB2, PAK3, PDGFB, PDGFC, PDK1, PF4, PITX2, PRKG1, RARG, SDCBP, SMN1, steroid, TGFB1, TIAM1 | Cell Morphology, Cellular Development, Cellular Movement | 12 |  |  |
| *Gene switching clusters (2 and 7)* | ACTL6B, APP, ATP7A, BCL11A, CLCN2, **CTSD**, HPRT1, MAPT, RBM9, **YWHAE** | ACTB, APPBP2, ATP, CACNL MAPPED, CDK5R2, CLIP1, Cu+, DNMBP, DOCK3, ELAVL4, ESR1, Esr1-Estrogen-Sp1, FPRL1 (includes EG:2358), Hsp70, ICMT, IL4, IL1B, Insulin, KLC1, KLC2, S100P, SMARCD1, TFCP2, THOP1 | Cell Morphology, Neurological Disease, Amino Acid Metabolism | 25 | Amyloid processing  Aryl hydrocarbon receptor signaling  Cell cycle: G2/M DNA damage checkpoint regulation | 1.45E-03  1.01E-02  4.22E-02 |
|  | CALM1, CHGB, **CTSD**, NEDD8, UBE2E3, **YWHAE** | AGER, CACNA1C, Calcineurin A, CAMK2G, CCL20, CHGA, CTSB, CUL2, EPB41, heme, IPO11, MARCKS (includes EG:4082), MT1E, MTPN, MYLK, NAE1, RAN, RCAN1, retinoic acid, S100B, SCARB1, SELL, SMPD1, SPHK1, TNF, TNFAIP2, UBE2M, VIP | Cell Death, Hematological Disease, Developmental Disorder | 13 |  |  |

^a^ A focus gene is an input gene that matched an annotated (based on human, mouse and rat) node in the Ingenuity knowledge base.

^b^ Associated nodes are intermediate molecules/complexes that are belonged to a network but are not an input gene.

^c^ Biological functions and/or disease annotations were based on all nodes (focus genes and associated nodes) in a novel molecular network.

^d^ A score of 2 indicates that there was a 1/100 chance that these genes were connected in a network due to random chance.

^e^ Enriched canonical pathways were determined based only on focus genes and the related *p* values were calculated using Fisher’s exact test.

**Table S4: Novel molecular networks, related biological functions/diseases and canonical pathways based on Ingenuity Pathway Analysis**

| **Gene clusters** | ***p* value** | **Canonical pathway^a^** | **Associated focus gene^b^** |
| --- | --- | --- | --- |
| *Embryonic specific gene clusters (1, 5 and 6)* | 1.83E-03  8.13E-03  2.23E-02 | Wnt/β-catenin signaling  P53 signaling  Tight junction signaling | *Sox4, Sfrp1 and Sox11*  *Cdk4 and Pcna*  *Cdk4 and Actb* |
| *Adult specific gene clusters (4, 8 and 10)* | 1.54E-05  9.60E-05  9.89E-04  1.30E-03  3.20E-03  7.68E-03  1.78E-02  3.03E-02  4.60E-02 | Synaptic Long Term Potentiation  Calcium Signaling  B Cell Receptor Signaling  cAMP-mediated Signaling  GM-CSF Signaling  Amyotrophic Lateral Sclerosis Signaling  Synaptic Long Term Depression  G-Protein Coupled Receptor Signaling  Xenobiotic Metabolism Signaling | *Gria3, Itpr1, Ppp3ca and Camk2a*  *Gria3, Itpr1, Ppp3ca and Camk2a*  *Egr1, Ppp3ca and Camk2a*  *Rgs4, Ppp3ca and Camk2a*  *Ppp3ca and Camk2a*  *Gria3 and Ppp3ca*  *Gria3 and Itpr1*  *Rgs4 and Camk2a*  *Gstm5 (Gstm1) and Camk2a* |
| *Gene switching clusters (2 and 7)* | 1.45E-03  1.01E-02  4.22E-02 | Amyloid processing  Aryl hydrocarbon receptor signaling  Cell cycle: G2/M DNA damage checkpoint regulation | *Mapt and App*  *Nedd8 and Ctsd*  *Ywhae* |

^a^ Enriched canonical pathways were determined based only on focus genes and the related *p* values were calculated based on Fisher’s exact test.

^b^ Focus gene is an input gene that matched an annotated (based on human, mouse and rat) node in Ingenuity knowledge base.

**Figure S4 Novel molecular network 1 (embryonic specific gene clusters 1, 5 and 6). Red coloured nodes=focus gene; non-coloured nodes = associated molecules; solid line=‘directly interact with’; dotted line=‘indirectly interact with’; A=‘activation’; I=‘inhibition’; E=‘regulating the expression’; PP=‘protein-protein interaction’; LO=‘regulating the localization’; PD=‘protein-DNA interaction’.**

**Figure S5 Novel molecular network 2 (embryonic specific clusters 1, 5 and 6). Red coloured nodes=focus gene; non-coloured nodes = associated molecules; solid line=‘directly interact with’; dotted line=‘indirectly interact with’; A=‘activation’; I=‘inhibition’; E=‘regulating the expression’; PP=‘protein-protein interaction’; LO=‘regulating the localization’; PD=‘protein-DNA interaction’.**

**Figure S6 Novel molecular network 3 (adult specific gene clusters 4, 8 and 10). Red coloured nodes=focus gene; non-coloured nodes = associated molecules; solid line=‘directly interact with’; dotted line=‘indirectly interact with’; A=‘activation’; I=‘inhibition’; E=‘regulating the expression’; PP=‘protein-protein interaction’; LO=‘regulating the localization’; PD=‘protein-DNA interaction’.**

**Figure S7 Novel molecular network 4 (adult specific gene clusters 4, 8 and 10). Red coloured nodes=focus gene; non-coloured nodes = associated molecules; solid line=‘directly interact with’; dotted line=‘indirectly interact with’; A=‘activation’; I=‘inhibition’; E=‘regulating the expression’; PP=‘protein-protein interaction’; LO=‘regulating the localization’; PD=‘protein-DNA interaction’.**

**Figure S8 Novel molecular network 5 (gene-switching clusters 2 and 7). Red coloured nodes=focus gene; non-coloured nodes = associated molecules; solid line=‘directly interact with’; dotted line=‘indirectly interact with’; A=‘activation’; I=‘inhibition’; E=‘regulating the expression’; PP=‘protein-protein interaction’; LO=‘regulating the localization’; PD=‘protein-DNA interaction’.**

**Figure S9 Novel molecular network 6 (gene-switching clusters 2 and 7). Red coloured nodes=focus gene; non-coloured nodes = associated molecules; solid line=‘directly interact with’; dotted line=‘indirectly interact with’; A=‘activation’; I=‘inhibition’; E=‘regulating the expression’; PP=‘protein-protein interaction’; LO=‘regulating the localization’; PD=‘protein-DNA interaction’.**

**SECTION D: TABULATION OF CELLULAR EXPRESSION DATA FOR VALIDATED DETS BASED ON INFORMATION IN PUBLICLY AVAILABLE EXPRESSION DATABASES**

Cellular expression of DETs were manually obtained from publicly available micrographs accessible from the Allen Institute for Brain Science website [32], Brain Gene Expression Map website [33] and Gene Expression Nervous System Atlas (GENSAT) website [34] and GenePaint website [35] (in descending order of database used). The cellular expression profiles of a small number of DETs not available on these databases were obtained from micrographs in the published literature. The expression intensity of each DET within the cerebral cortex was classified using a scale from 0-2 (Table S5). The expression intensities of one DET should not be compared to another DET based on this scale. However, the relative cellular expression of a DET within the cortical layers of a single timepoint is relatively comparable.

Classification of DETs into neuron (N), glia (G) or both (B) group was based on publicly available expression micrographs deposited in the Gene Expression Nervous System Atlas (GENSAT) website [34], IPA knowledgebase [118] and PubMed literature search (by using a combination of the following keywords “gene name”, “neuron”, “glial”, cerebral cortex”). Only gene expression reported within the cerebral cortex of either mouse or rat was taken into consideration.

**Table S5 : Cellular expression of validated DETs based on publicly available micrographs or expression databases.**

| **No.** | **Gene** | **Embryonic development (E15.5)^a^** | | | | | | **Afterbirth** | | | | | | | | | | | | | | **Group (N, G or B)^e^** |
| --- | --- | --- | --- | --- | --- | --- | --- | --- | --- | --- | --- | --- | --- | --- | --- | --- | --- | --- | --- | --- | --- | --- |
|  |  |  |  |  |  |  |  | **Postnatal^b^** | | | | | | | **Adult^c^** | | | | | | |  |
|  |  | **SVZ/VZ** | **IZ** | **SP** | **CP** | **MZ** | **REF** | **6** | **5** | **4** | **2 or 3** | **1** | **CT ^d^** | **REF** | **6** | **5** | **4** | **2 or 3** | **1** | **CT ^d^** | **REF** |  |
| 1 | Camk2a | 2 | 1 | 1 | 1 | 1 | [33] | 1 | 1 | 1 | 2 | 1 | N | [33, 34] | 1 | 1 | 1 | 2 | 1 | N | [32, 34] | N |
| 2 | Egr1 | 1 | 1 | 2 | 1 | 1 | [33, 34] | 1 | 1 | 1 | 1 | 1 | N,G | [33, 34] | 2 | 1 | 2 | 2 | 1 | N | [32, 34] | B |
| 3 | Plp1 | 0 | 0 | 0 | 0 | 0 | [33] | 0 | 0 | 0 | 0 | 0 | - | [33] | 2 | 1 | 1 | 1 | 1 | G | [32, 52] | G |
| 4 | Camk2n1 | 0 | 0 | 0 | 1 | 1 | IH | 1 | 1 | 1 | 1 | 1 | - | IH | 0 | 0 | 1 | 2 | 1 | N | [44],IH | N |
| 5 | Cryab | 0 | 0 | 0 | 0 | 0 | [33, 34] | 2 | 2 | 1 | 0 | 0 | - | [33] | 2 | 2 | 2 | 1 | 1 | G | [32, 34] | G |
| 6 | Nrgn | 0 | 0 | 0 | 0 | 0 | [32] | 0 | 0 | 1 | 1 | 1 | N | [33, 34] | 2 | 2 | 2 | 2 | 1 | N | [3] | N |
| 7 | BQ176089 | - | - | - | - | - | - | - | - | - | - | - | - | - | - | - | - | - | - | - | - | B |
| 8 | Sept4 | 2 | 1 | 1 | 2 | 1 | [33] | 1 | 1 | 1 | 1 | 1 | - | [33] | 1 | 1 | 1 | 1 | 1 | G | [32, 34] | G |
| 9 | Sncb | 1 | 1 | 2 | 2 | 1 | [33] | 1 | 1 | 1 | 1 | 1 | - | [33] | - | - | - | - | - | N | [45] | N |
| 10 | Nptxr | - | - | - | - | - | - | - | - | - | - | - | - | - | - | - | - | - | - | G | [46] | G |
| 11 | Ppp1r1b | 0 | 1 | 1 | 2 | 1 | [33, 34] | 1 | 1 | 1 | 1 | 0 | N | [33, 34] | 2 | 1 | 1 | 2 | 1 | N | [32, 34] | N |
| 12 | Gstm1 | 1 | 0 | 0 | 0 | 0 | [33] | - | - | - | - | - | - | - | 0 | 1 | 1 | 1 | 0 | N,G | [32, 43] | B |
| 13 | Chgb | 0 | 1 | 1 | 2 | 1 | [33, 34] | 2 | 2 | 2 | 1 | 0 | N,G | [33, 34] | 1 | 1 | 1 | 1 | 1 | N,G | [32, 34] | B |
| 14 | Itpr1 | 1 | 1 | 2 | 2 | 1 | [33] | 1 | 1 | 1 | 2 | 1 | - | [33] | 1 | 1 | 1 | 2 | 1 | N | [32, 47] | N |
| 15 | Rgs4 | 0 | 1 | 2 | 1 | 1 | [34] | 1 | 1 | 1 | 1 | 1 | N | [34] | 1 | 2 | 1 | 2 | 0 | N | [32, 34] | N |
| 16 | AK139402 | - | - | - | - | - | - | - | - | - | - | - | - | - | - | - | - | - | - | - | - | B |
| 17 | Tspan7 | 2 | 2 | 2 | 1 | 1 | [33] | 1 | 1 | 2 | 2 | 1 | G | [33, 34] | 1 | 1 | 1 | 1 | 1 | G | [32, 34] | G |
| 18 | Sirpa | 2 | 1 | 1 | 2 | 2 | [33] | 1 | 1 | 1 | 1 | 1 | N,G | [33, 34] | 1 | 1 | 1 | 1 | 0 | G | [32, 34] | B |
| 19 | AU258168 | - | - | - | - | - | - | - | - | - | - | - | - | - | - | - | - | - | - | - | - | B |
| 20 | Ppp3ca | 0 | 0 | 0 | 0 | 0 | [33] | 1 | 1 | 1 | 1 | 0 | - | [32, 33] | 1 | 1 | 1 | 2 | 0 | N | [32, 48] | N |
| 21 | App | 0 | 0 | 0 | 1 | 1 | [35] | - | - | - | - | - | - | - | 1 | 1 | 1 | 1 | 1 | N,G | [32, 46, 55] | B |
| 22 | Hprt1 | 1 | 1 | 1 | 1 | 1 | [33] | 1 | 1 | 1 | 1 | 0 | - | [33] | 1 | 1 | 1 | 1 | 0 | N | [32, 56] | N |
| 23 | Gria3 | - | - | - | - | - | - | - | - | - | - | - | - | - | 1 | 1 | 1 | 1 | 1 | N,G | [32, 46, 49, 50] | B |
| 24 | Calm1 | 2 | 1 | 1 | 1 | 1 | [33] | 1 | 1 | 1 | 1 | 0 | - | [33] | 1 | 1 | 1 | 1 | 1 | N | [32, 58] | N |
| 25 | Ctsd | 2 | 1 | 1 | 1 | 1 | [33] | 1 | 1 | 1 | 1 | 0 | - | [33] | 1 | 1 | 1 | 1 | 1 | N,G | [32, 46, 57] | B |
| 26 | Mbp | 0 | 0 | 0 | 0 | 0 | [33] | 1 | 1 | 0 | 0 | 0 | - | [33] | 1 | 1 | 1 | 1 | 1 | G | [32, 51] | G |
| 27 | Ppap2b | 1 | 1 | 0 | 0 | 0 | [35] | - | - | - | - | - | - | - | 1 | 1 | 1 | 1 | 1 | G | [32, 46] | G |
| 28 | AK138272 | - | - | - | - | - | - | - | - | - | - | - | - | - | - | - | - | - | - | - | - | B |
| 29 | AK140219 | - | - | - | - | - | - | - | - | - | - | - | - | - | - | - | - | - | - | - | - | B |
| 30 | Clcn2 | 1 | 1 | 1 | 1 | 1 | [33] | 1 | 1 | 1 | 1 | 0 | - | [33] | 1 | 2 | 1 | 1 | 0 | - | [32] | B |
| 31 | AK154943 | - | - | - | - | - | - | - | - | - | - | - | - | - | - | - | - | - | - | - | - | B |
| 32 | Als2cr2 | 1 | 1 | 1 | 2 | 1 | [33] | 1 | 1 | 1 | 1 | 0 | - | [33] | 0 | 0 | 0 | 0 | 0 | - | [32] | - |
| 33 | Nedd8 | 2 | 1 | 1 | 2 | 1 | [33] | 1 | 1 | 1 | 1 | 0 | N | [33, 34] | 1 | 1 | 1 | 2 | 1 | N | [32-34] | N |
| 34 | Ywhae | 2 | 1 | 1 | 2 | 1 | [33] | 1 | 1 | 1 | 2 | 1 | - | [33] | 1 | 1 | 1 | 1 | 1 | N | [32, 33, 59] | N |
| 35 | Ube2e3 | 0 | 0 | 0 | 0 | 0 | [35] | - | - | - | - | - | - | - | 0 | 0 | 0 | 0 | 0 | - | [32] | B |
| 36 | Rps4x | 2 | 1 | 1 | 1 | 1 | [33] | 1 | 1 | 1 | 1 | 1 | - | [33] | 1 | 1 | 1 | 1 | 1 | - | [32, 33] | - |
| 37 | Pcna | 2 | 1 | 0 | 0 | 0 | [36] | - | - | - | - | - | - | - | 0 | 0 | 0 | 0 | 0 | N,G | [31, 32] | - |
| 38 | Cdkn1c | 0 | 1 | 0 | 1 | 1 | [33] | 0 | 0 | 0 | 0 | 0 | - | [33] | 0 | 0 | 0 | 1 | 0 | N | [32, 37] | - |
| 39 | 5031439G07Rik | 0 | 0 | 0 | 1 | 1 | [35] | - | - | - | - | - | - | - | 1 | 2 | 1 | 1 | 0 | - | [32] | - |
| 40 | Bcl11a | 1 | 1 | 1 | 2 | 0 | [54] | - | - | - | - | - | - | - | 1 | 1 | 1 | 1 | 0 | N | [32, 60] | N |
| 41 | H2afy | 2 | 1 | 1 | 2 | 1 | [33] | 1 | 1 | 1 | 1 | 1 | - | [33] | 1 | 1 | 1 | 1 | 0 | - | [32] | - |
| 42 | Neurod1 | 0 | 2 | 1 | 1 | 2 | [34] | 1 | 1 | 1 | 1 | 1 | N | [34] | 0 | 0 | 0 | 0 | 0 | N | [32, 34] | - |
| 43 | Marcks | 2 | 1 | 1 | 2 | 1 | [33] | 1 | 1 | 1 | 2 | 1 | - | [33] | 1 | 1 | 1 | 1 | 1 | N,G | [32, 38, 39] | - |
| 44 | Rbm9 | 1 | 1 | 1 | 2 | 1 | [35] | - | - | - | - | - | - | - | 1 | 1 | 1 | 1 | 1 | N | [32, 61] | N |
| 45 | Wsb1 | 1 | 2 | 1 | 1 | 1 | [35] | - | - | - | - | - | - | - | 1 | 1 | 1 | 1 | 1 | - | [32] | - |
| 46 | Btg1 | 2 | 0 | 1 | 1 | 1 | [33] | 1 | 1 | 1 | 2 | 1 | - | [33] | 1 | 1 | 1 | 2 | 1 | - | [32] | - |
| 47 | Robo1 | 0 | 0 | 0 | 1 | 0 | [35] | - | - | - | - | - | - | - | 0 | 1 | 0 | 0 | 0 | N | [32, 40] | - |
| 48 | AA122503 | - | - | - | - | - | - | - | - | - | - | - | - | - | - | - | - | - | - | - | - | - |
| 49 | Cdk4 | 2 | 1 | 1 | 1 | 1 | [33] | 1 | 1 | 1 | 1 | 0 | N,G | [33, 34] | 1 | 1 | 1 | 1 | 1 | G | [32, 34] | - |
| 50 | Sfrp1 | 2 | 0 | 1 | 1 | 1 | [33] | 1 | 1 | 1 | 1 | 1 | N,G | [33, 34] | 1 | 1 | 1 | 2 | 1 | N,G | [32, 34] | - |
| 51 | Ezh2 | 2 | 2 | 1 | 1 | 1 | [35] | - | - | - | - | - | - | - | 0 | 0 | 0 | 0 | 0 | G | [32, 41] | - |
| 52 | Zfp57 | 0 | 0 | 0 | 2 | 1 | [35] | - | - | - | - | - | - | - | 0 | 0 | 0 | 1 | 0 | - | [32] | - |
| 53 | Dcx | 1 | 1 | 1 | 2 | 1 | [33] | 1 | 1 | 1 | 1 | 1 | N | [33, 34] | 0 | 0 | 0 | 0 | 0 | N | [32, 34] | - |
| 54 | Sox11 | 1 | 2 | 1 | 2 | 1 | [33] | 1 | 1 | 1 | 1 | 1 | N | [33, 34] | 0 | 0 | 0 | 0 | 0 | N | [32, 34] | - |
| 55 | Dr1 | 1 | 1 | 1 | 2 | 1 | [42] | - | - | - | - | - | - | - | 0 | 1 | 1 | 1 | 0 | N | [32, 42] | - |
| 56 | Zswim4 | - | - | - | - | - | - | - | - | - | - | - | - | - | 0 | 1 | 1 | 1 | 0 | - | [32] | - |
| 57 | Actl6b | 0 | 1 | 1 | 2 | 1 | [35] | - | - | - | - | - | - | - | 1 | 1 | 1 | 1 | 1 | - | [32] | B |
| 58 | Atp7a | 2 | 1 | 1 | 1 | 1 | [35] | - | - | - | - | - | - | - | 0 | 0 | 0 | 0 | 0 | N,G | [32, 53] | B |
| 59 | Hmgb3 | 1 | 1 | 1 | 1 | 0 | [35] | - | - | - | - | - | - | - | 0 | 1 | 1 | 1 | 0 | - | [32] | B |
| 60 | BQ177886 | - | - | - | - | - | - | - | - | - | - | - | - | - | - | - | - | - | - | - | - | - |
| 61 | Actb | 1 | 2 | 1 | 2 | 1 | [33] | 1 | 1 | 1 | 2 | 1 | - | [33] | 1 | 1 | 1 | 2 | 1 | N,G | [32] | - |
| 62 | Cand2 | 2 | 1 | 1 | 1 | 1 | [35] | - | - | - | - | - | - | - | 1 | 1 | 1 | 1 | 1 | - | [32] | - |
| 63 | Sox4 | 1 | 1 | 1 | 2 | 1 | [33] | 1 | 1 | 1 | 2 | 1 | N,G | [33, 34] | 1 | 1 | 1 | 1 | 1 | N | [33, 34] | - |
| 64 | Csrp2 | 0 | 1 | 0 | 1 | 0 | [35] | - | - | - | - | - | - | - | 0 | 0 | 0 | 0 | 0 | - | [32] | - |
| 65 | Mapt | 0 | 1 | 2 | 2 | 2 | [33] | 1 | 1 | 1 | 1 | 1 | N | [33, 34] | 1 | 1 | 1 | 1 | 1 | N | [32, 34] | N |
| 66 | Blcap | 0 | 0 | 0 | 0 | 1 | [35] | - | - | - | - | - | - | - | 1 | 1 | 1 | 1 | 0 | - | [32] | - |
| 67 | CD802535 | - | - | - | - | - | - | - | - | - | - | - | - | - | - | - | - | - | - | - | - | - |
| 68 | Tmsb4x | 1 | 2 | 1 | 2 | 1 | [33] | 1 | 1 | 1 | 2 | 1 | - | [33] | 1 | 1 | 2 | 2 | 1 | N | [32] | - |

^a^ Based on E15.5 micrographs from [32-34] or E14.5 micrographs from [35].

^b^ Based on P4 [32] or P7 [33] micrographs.

^c^ Based on P56 micrographs from [32, 33].

^d^ Based on micrographs from [34], IPA knowledgebase [118] and published literature. Only expression datasets generated from cortical neurons or glial cells of mouse and rat were considered.

^e^ Some of the DETs were not classified into the N-, G- or B-groups because they do not belong to the IPA Networks 3 to 6 in IPA analysis.

Note : IH denotes in-house experiment. A dash (-) denotes no expression information was found. The intensity of expression values range from 0-2 where 0 denotes ‘no expression’, 1 denotes ‘relatively higher expression’ and 2 denotes ‘obvious expression’.


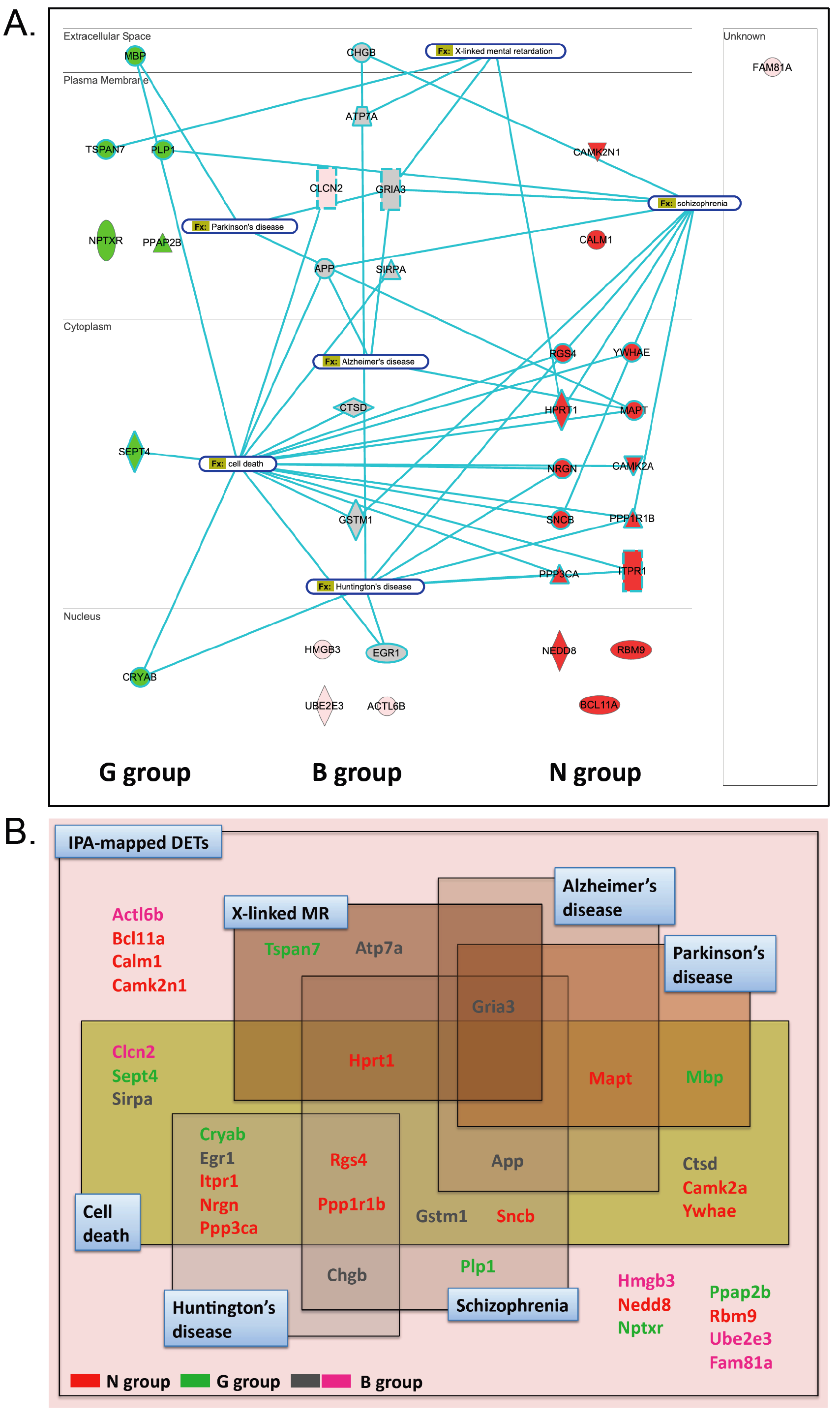


**Figure S10 (A) IPA analysis of the qPCR-validated DETs from Networks 3 to 6 and their associated neurological disorders and cell death process. DETs were classified into three groups according to the origin of their expression; only expressed in cortical neurons (N-group), in cortical glia (G-group) or in both cell types (B-group). DETs without known cellular expression data were classified in B-group. Red coloured nodes = N-group DETs, green coloured nodes = G-group DETs and both grey and pink (DETs without cellular expression data) coloured nodes = B-group DETs. (B) Simplified Venn diagram to illustrate the association between N-, G- and B-group DETs according to (A). X-linked MR denotes ‘X-linked mental retardation’.**

**SECTION E: ANALYSIS OF THE *SOX4* GENOMIC CLUSTER**

**Tel**

**VZ**

**CP**

**R**

**C**

**IZ**

**CP**

**OB**

**Figure S11 Regionalization of *Sox4* transcripts. A-F are sagittal sections obtained from various developmental stages of the mouse cerebral cortex. Arrowheads show brain regions with higher *Sox4* sense expression. Both E15.5 and E17.5 brain sections show regionalized expression of *Sox4* in the cerebral cortex. Tel denotes telencephalon; VZ denotes ventricular zone; CP denotes cerebral cortex; C denotes caudal region; R denotes rostral region; IZ denotes intermediate zone; OB denotes olfactory bulb.**


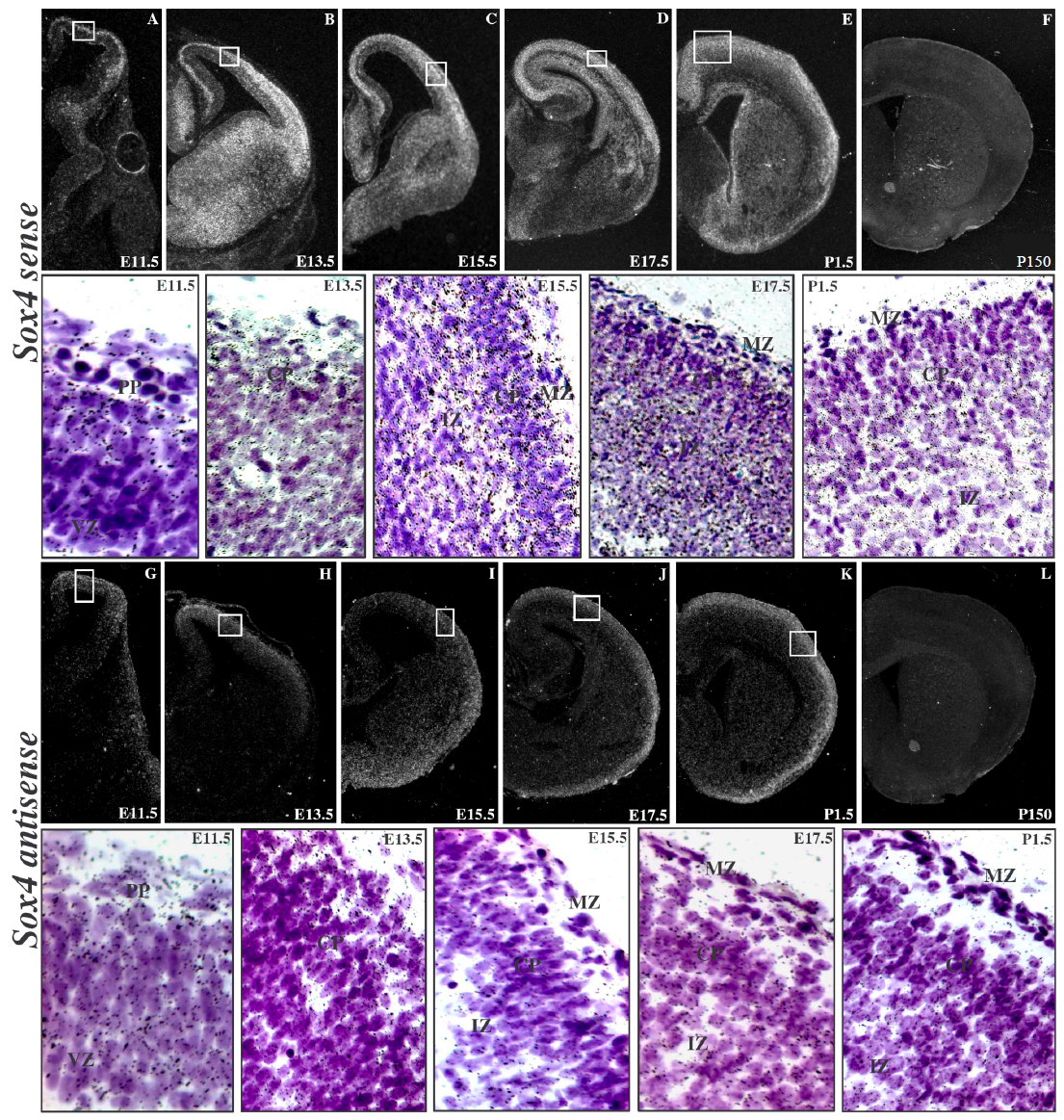


**Figure S12 ISH of *Sox4* transcripts in E11.5 to P150 mouse brains. A-F show the expression of the sense transcript for *Sox4*. G-L show the expression of antisense transcripts for *Sox4*. Bright field micrographs show high magnification snapshots of the corresponding dark field micrographs. All micrographs were taken from coronal sections. Silver grains are seen as black dots under bright field. The silver grains confirm that the expression of both the sense and antisense transcripts are not due to background noise. PP=primordial plexiform layer; SVZ=subventricular zone; IZ=intermediate zone; CP=cortical plate; MZ=marginal zone.**

**
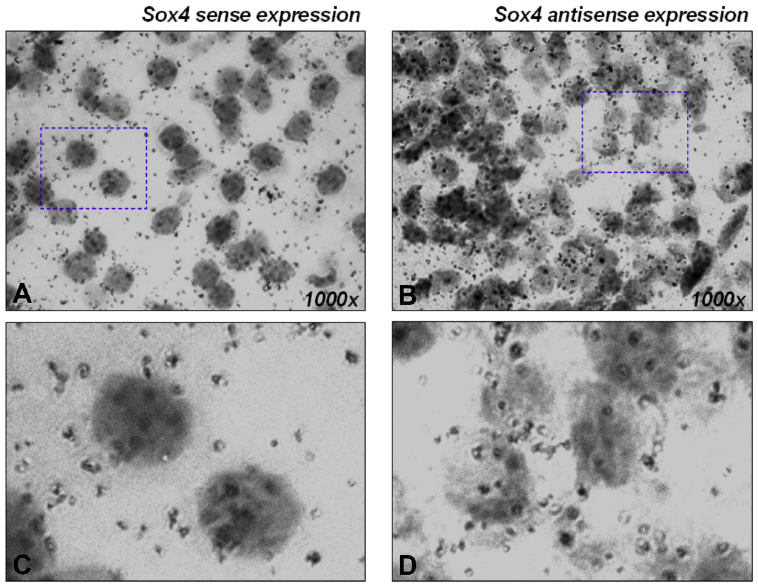
**

**Figure S13 A high magnification examination of *Sox4* transcript ISH. A and C show the expression of *Sox4* sense transcripts that are clearly detectable in both the nucleus and cytoplasm. B and D show the expression of *Sox4* antisense transcripts that are predominantly localized in the nucleus. All micrographs were taken of the P1.5 cerebral cortex. Both C and D are the enlarged micrographs corresponding to A and B, respectively.**

**
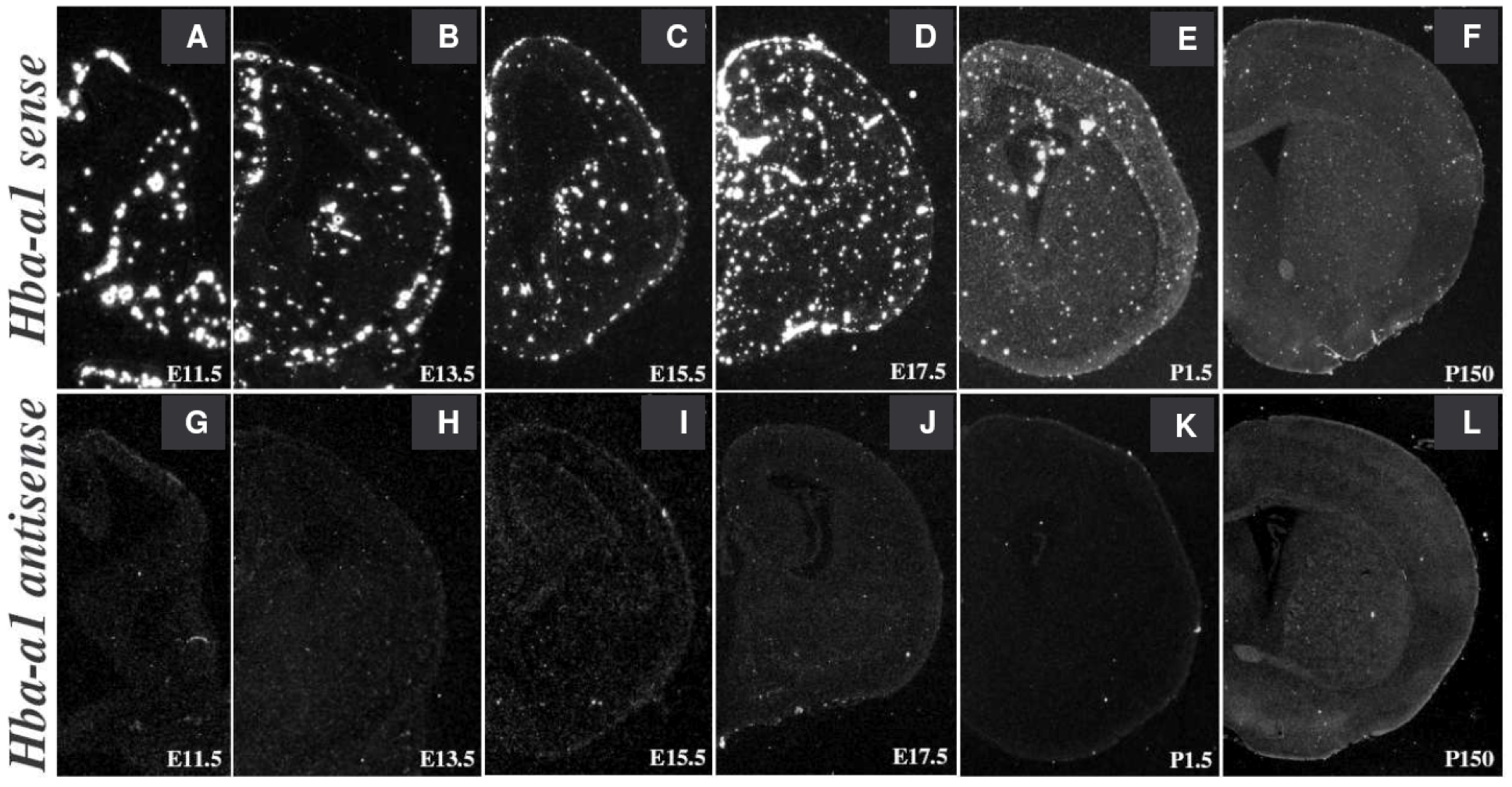
**

**Figure S14 ISH of the *Hba-a1* transcript in E11.5 to P150 mouse brains. A-F show the expression of the sense transcript whereas G-L represent the expression of the antisense transcript for *Hba-a1*. Sense expression of *Hba-a1* appeared ‘dotted’ because the transcript was expressed only by specific haematopoetic cells such as reticulocytes that clump within vascular compartments. There was no ‘dotted’-like appearance in adult sections because adult mice were perfused with saline prior to fixation. We found no observable antisense expression for *Hba-a1* at any developmental stages (10F.1-F.6). Therefore, *Hba-a1* is not only a good candidate control gene for the spatio-temporal study of gene expression, it also provides quality assurance on technical issues such as tissue preparation.**

**Table S6: Mapped Paired End Di-tags (PETs) at the *Sox4* gene locus.**

| **FANTOM PET ID** | **Chromosome** | **Start position^a^** | **End position^b^** | **Strand** | **Size (nt)^c^** |
| --- | --- | --- | --- | --- | --- |
| 154342 | chr13 | 29043154 | 29043961 | + | 807 |
| 153327 | chr13 | 29042250 | 29045096 | + | 2846 |
| 87809 | chr13 | 29041180 | 29045566 | + | 4386 |
| 31530 | chr13 | 29041285 | 29044501 | + | 3216 |
| 32641 | chr13 | 29041310 | 29043229 | + | 1919 |
| 154138 | chr13 | 29043742 | 29045566 | + | 1824 |

^a^ Start position for the FANTOM PET left tag.

^b^ End position for the FANTOM PET right tag.

^c^ Size of the predicted transcript based on the left and right PETs presuming no splicing has occured.

^a,b^ FANTOM PET sequences were obtained from Ensembl website [64]. The ditag of PolyA+ RNA libraries were originally downloaded from the FANTOM consortium site and processed using code available in <http://www.ensembl.org/Mus_musculus/ditags/FANTOM_GSC_PET.html>.

**Table S7: miRNAs, which target *Sox4 sense* transcripts.**

| **miRNA** | **Targeted position^a^** | | | |
| --- | --- | --- | --- | --- |
|  | **Chromosome** | **Start position** | **End position** | **Strand** |
| mmu-miR-324-5p | chr13 | 29042673 | 29042695 | - |
| mmu-miR-331-3p | chr13 | 29042520 | 29042542 | - |
| mmu-miR-466a-3p | chr13 | 29041141 | 29041163 | - |
| mmu-miR-702 | chr13 | 29043538 | 29043560 | - |

^a^ Analysis was based on the miRanda 3.0 prediction created on 2007-11-1, which was downloaded from [103]. Only mouse miRNAs are shown. The targeted positions were re-mapped onto the NCBI build 37.1 mouse assembly database.

**Figure S15 UCSC genome browser view of miRNAs, which target *Sox4* sense transcripts with corresponding SAGE tags and mouse mRNAs.**

**SECTION F: ANALYSIS OF THE *SOX11* GENOMIC CLUSTER**

***Genomic cluster at the Sox11 locus***

There were 14 SAGE tags situated within the *Sox11* locus (Figure S16-A). Of these tags, 10 were located within the *Sox11* canonical transcript. Only two DETs within the canonical transcript were identified by 3’ RACE Southern blotting: sox11_tag11 and sox11_tag12. According to the SAGE analysis, both DETs were expressed highly in embryonic stages of development compared to P1.5 or Ad (Figure S16-B). RT-qPCR analysis using two different assays near to these tags confirmed the embryonic specific expression of sox11_tag11 and sox11_tag12 at P1.5 (with fold changes of -2.50 and -3.32 respectively; *p*<0.0001) and P150 (with fold changes of -140.10 and -139.80 respectively; *p*<0.0001) compared to E15.5 (Figure S16-C). In agreement with the SAGE analysis, RT-qPCR showed consistently higher expression of the sox11_tag12 than the sox11_tag11 at all stages. Figure S16-D shows the strand specific 3’ RACE-Southern blotting analysis of *Sox11*. The analysis confirms the existence of the multiple overlapping sense transcripts (Figure S16-D.1-D.3) within the *Sox11* canonical transcript based on the SAGE expression profile. Figure S16-D.3 shows the sox11_tag11 amplicons, which range from 0.65kb-1.35kb. Amplicon sizes beyond 0.75kb are most likely to indicate the presence of alternative transcripts that contribute to the tags situated beyond the canonical mRNA; sox11_tag6, sox11_tag7 and sox11_tag9. In all cases, these transcripts were embryonic specific and expressed during cerebral cortical development. We also confirmed the existence of the sox11_tag12, which showed consistent expression throughout cortical development with only mild differences in expression between E15.5, P1.5 and P150. The same analysis with antisense specific probes confirmed the expression of antisense messages (sox11_tag16 and sox11_tag17) within this genomic locus (Figure S16-D.4). Sox11_tag16 and sox11_tag17 were expressed during the embryonic stages of development, and not at P1.5 or P150. The controls genes used in the 3’ RACE-Southern blotting analysis were the same genes used in the *Sox4* analysis, *Psmb2* and *Hmbs* (Figure S16-D.5-D.6). In addition, we also performed Northern analysis on *Sox11* using a ~1.0kb double stranded DNA probe spanning both sox11_tag16 and sox11_tag17. The Northern analysis showed only two prominent transcript variants ranging between ~7.0-8.0kb. Northern analysis of *Sox11* (Figure S16-E) may not reflect the actual transcript variants that exist in the cluster for various reasons, these include: the probe does not span the relevant overlapping transcripts, or the overlapping region is limited.

**Figure S16 Diagram A shows the UCSC genome browser at the *Sox11* genomic locus. SAGE tags are found in both directions within the *Sox11* reference gene. B shows the SAGE expression profiles for each tag in the *Sox11* locus. C shows the RT-qPCR validations of selected DETs at various loci within the *Sox11* canonical gene whereas D shows the 3’ RACE-Southern blotting analysis. D.1-D.3 show the amplification of *Sox11* sense transcripts whereas D.4 represents the amplifications of *Sox11 a*ntisense transcripts. Tags with asterisks (*) are DETs. Both D.5 and D.7 are positive controls exclusively generated from the sense strand of *Psmb2* and *Hmbs* endogenous genes. The corresponding D.6 and D.8 show the antisense expression (negative control) of *Psmb2* and *Hmbs*, respectively. E shows a Northern analysis of total RNA isolated from pooled mouse cerebral cortices (n=7).**

**
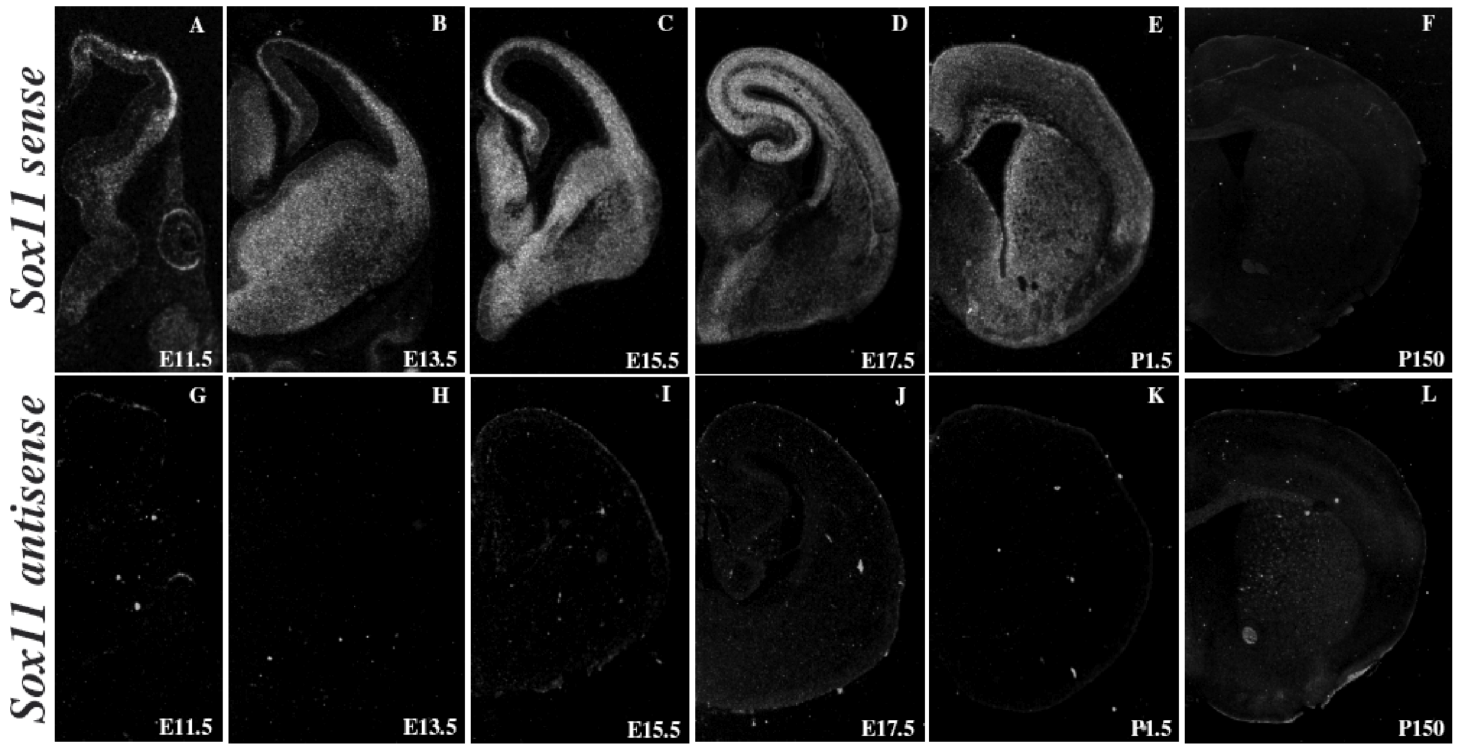
**

**Figure S17 ISH of *Sox11* transcripts from E11.5 to P150 mouse brains. A-F show the expression of sense transcripts for *Sox11*. G-L show the ISH of antisense transcripts for *Sox11*. (A) At E11.5, *Sox4* sense transcripts were confined to the primordial plexiform layer. (B-E) From E13.5 to P1.5, the sense transcripts were expressed throughout the cortical plate. Expression of sense transcripts at the subventricular zones was observed at E17.5 (D) and P1.5 (E) only. There was no observable expression of the sense transcript during the adult stage (F). The expression of the antisense transcript was not detected in all ISH sections (G-L).**

**Table S8: Mapped Paired End Di-tags (PETs) at the *Sox11* gene locus.**

| **FANTOM PET ID** | **Chromosome** | **Start position^a^** | **End position^b^** | **Strand** | **Size (nt)^c^** |
| --- | --- | --- | --- | --- | --- |
| 43777 | chr12 | 28021261 | 28023139 | + | 1878 |
| 43643 | chr12 | 28021321 | 28022447 | + | 1126 |
| 43286 | chr12 | 28021782 | 28023532 | + | 1750 |
| 43573 | chr12 | 28022508 | 28023136 | + | 628 |
| 43301 | chr12 | 28022803 | 28025440 | + | 2637 |
| 43174 | chr12 | 28023092 | 28024493 | + | 1401 |
| 168702 | chr12 | 28023106 | 28024669 | + | 1563 |

^a^ Start position for the FANTOM PET left tag.

^b^ End position for the FANTOM PET right tag.

^c^ Size of the predicted transcript based on the left and right PETs presuming no splicing of transcript.

^a,b^ FANTOM PET sequences were obtained from EnsEMBL project website [64]. The ditag of PolyA+ RNA libraries were originally downloaded from the FANTOM consortium site and processed using code available in <http://www.ensembl.org/Mus_musculus/ditags/FANTOM_GSC_PET.html>.

**Table S9: miRNAs, which target *Sox11* sense transcripts.**

| **miRNA** | **Targeted position^a^** | | | |
| --- | --- | --- | --- | --- |
|  | **Chromosome** | **Start position** | **End position** | **Strand** |
| mmu-let-7g* | chr12 | 28025175 | 28025196 | - |
| mmu-miR-136* | chr12 | 28025967 | 28025988 | - |
| mmu-miR-207 | chr12 | 28026014 | 28026035 | - |
| mmu-miR-20b* | chr12 | 28025454 | 28025475 | - |
| mmu-miR-337-5p | chr12 | 28025682 | 28025701 | - |
| mmu-miR-343 | chr12 | 28025708 | 28025727 | - |
| mmu-miR-378* | chr12 | 28026044 | 28026065 | - |
| mmu-miR-409-5p | chr12 | 28025311 | 28025333 | - |
| mmu-miR-453 | chr12 | 28025325 | 28025348 | - |
| mmu-miR-540-5p | chr12 | 28025633 | 28025652 | - |
| mmu-miR-151-5p | chr12 | 28026067 | 28026088 | - |

^a^ Analysis was based on the miRanda 3.0 prediction created on 2007-11-1, which was downloaded from [103]. Only mouse miRNAs are shown. The targeted positions were re-mapped onto the NCBI build 37.1 mouse assembly database.

**Figure S18 UCSC genome browser view for miRNAs, which target *Sox11* sense transcripts and corresponding SAGE tags and mouse mRNAs.**

**SECTION G: SUPPLEMENTARY MATERIALS AND METHODS**

*Total RNA isolation and first strand cDNA synthesis*: An additional DNase I (Qiagen) treatment step was added prior to recovery of total RNA to remove traces of genomic DNA (gDNA) contamination. The concentration and quality of all isolated total RNA was determined using the 2100 Bioanalyzer (Agilent Technologies). Only total RNAs with an RNA Integrity Number [119] greater than 8.0 were considered for subsequent RT-qPCR analysis.

*Primer design and RT-qPCR*: The following parameters were applied to all primers designed; length between 18-27bp, GC% between 30-70, Tm between 59-60 and amplicon size between 50-250bp. All RT-qPCR reactions were prepared in 10μl volumes in a 384-well plate format with 1X LC480 Master Probe Mix (Roche Diagnostics), 250nM of forward and reverse primers (GeneWorks or Bioneer Corporation) and 100nM of Universal ProbeLibrary probe (Roche Diagnostics). RT-qPCRs were performed using the LightCycler® 480 System (Roche Diagnostics) with a pre-denaturing step of 95^o^C for 10 minutes, and 45 cycles of 95^o^C (10 seconds), 60^o^C (30 seconds) and 72^o^C (10 seconds) followed by a cooling step at 40^o^C for 1 minute.

*Relative quantification using a standard curve method*: The following criteria were adopted to define a successful PCR assay; PCR efficiency between 80-120%, R-squared>0.985 and a minimum of two successful housekeeping genes in each run. Based on a successful standard curve, the amount of starting RNA of both target and reference genes were calculated as a linear function of logarithmic concentration and Cp. All estimated starting amounts were in arbitrary values.

*Strand specific RT-PCR*: PCR amplifications were carried out in using FastStart PCR High Fidelity System (Roche Diagnostics) according to manufacturer’s protocol. The cycling parameters were as follows; a pre-denaturing step at 95^o^C for 10 minutes, and 35 cycles of 95^o^C (30 seconds), 60^o^C (30 seconds) and 72^o^C (1 minute) followed by an additional extension at 72^o^C for 7 minutes.

*3’ Rapid Amplification of cDNA Ends (RACE)*: PCR amplifications were carried out in 40µl reaction using FastStart PCR High Fidelity System (Roche Diagnostics) at 95^o^C (10 minutes) initial denaturation, and 40 cycles of 95^o^C (30 seconds), 60^o^C (1 minute) and 72^o^C (1 minute) followed by a final extension at 72^o^C for 7 minutes (if necessary).

*Southern blotting analysis*: Prehybridization of blots was carried out at 42^o^C in 125μl /cm^2^ Rapid-Hyb buffer (GE Healthcare) for 20 minutes. Hybridizations were performed in the same solution and incubated for 2 hours at 42^o^C with 1-3x10^6^ dpm/ml of 5’ end-labelled probe. Membranes were initially washed with 5X SSC (with 0.1% v/v SDS) for 20 minutes at 37^o^C followed by 1X SSC (with 0.1% v/v SDS) for 15 minutes with a gradual increase in washing temperature up to 65^o^C. A final wash (if needed) was performed using fresh 1X SSC (with 0.1% v/v SDS) at 65^o^C for an additional 15 minutes.

Synthetic oligonucleotides were 5’ end-labelled using T4 polynucleotide kinase (T4-PNK) (Promega) and [γ-^32^P]ATP (Amersham). The reactions were prepared in a 25µl volume with 20U of T4-PNK, 0.05mCi of [γ-^32^P]ATP and 2.5ul of 10X kinase buffer (Promega). The reactions were incubated for 20 minutes at 37^o^C before 2ul of 0.5M EDTA was added to stop the reaction. Unincorporated radionucleotides were separated from the labelled probe through a Sephadex G-25 column. See SI-7 for detailed primer sequences and oligonucleotides used for detection.

*In situ RNA hybridization* *(ISH)*: All dissected embryonic and postnatal brains were fixed for 24-48 hours in cold 4% paraformaldehyde without cardiac perfusion. For E11.5 and E13.5, whole embryo heads were fixed. For adult mice, cardiac perfusion was performed using 4% paraformaldehyde prior to dissection. Dissected adult brains were then fixed in cold 4% paraformaldehyde for 48 hours. Brain sections were dewaxed, rehydrated through graded concentrations of ethanol, incubated for 30 minutes at room temperature in 10mg/ml proteinase K, followed by additional fixation in 4% paraformaldehyde for 10 minutes, then dehydrated through graded concentrations of ethanol. Pre-processed brain sections were air-dried, then hybridization solution containing 5x10^5^ cts/minute/µl *in vitro* transcribed cRNA probes (Riboprobe® In Vitro Transcription System Kit by Promega) was placed over the section. Slides were incubated overnight at 56^o^C in a formamide-humidified chamber and then washed as described previously [123]. Washed slides were exposed to Kodak NTB-2 (Kodak) emulsion at 4^o^C for 7-21 days and then developed using Kodak Professional Developer Characteristics D-19 (Kodak).

*Strand-specific RT-qPCR:* Total RNA was extracted from harvested organs using the TRIzol®’s reagent (Invitrogen) according to the manufacturer’s protocol. To avoid genomic DNA contamination, all isolated total RNA was treated with the recombinant DNAse I enzyme provided by the DNA-*free*^TM^ kit (Applied Biosystems) according to the manufacturer’s protocol. The concentration and purity of all isolated total RNA was determined using the Nanodrop^TM^ 1000 spectrophotometer (Thermo Scientific). First strand cDNA synthesis was carried out using strand-specific primers followed by qPCR analysis as described in above.

*Embryonic neural stem cells grown as neurospheres*: A neuroculture complete medium consists of: 1X NEUROBASAL™ Medium (Cat number: 21103-049, Invitrogen) containing 2% (v/v) 50X B-27 serum-free supplement (Cat number: 17504-044, Invitrogen), 1% (v/v) 200mM L-glutamine, 1% (v/v) 200mM Glutamax (Cat number: 35050-061, Invitrogen), 100 units/ml penicillin, 100 µg/ml streptomycin, 20ng/ml EGF (BD Biosciences) and 20ng/ml bFGF (R & D Systems).

*P19 embryonal carcinoma (EC) cells*: A P19GM complete medium consists of: Minimum Essential Medium Alpha (alpha-MEM; Cat number: 12571-071; Invitrogen) supplemented with 10% (v/v) heat-inactivated fetal calf serum (FCS; Cat number: 10438-026; Invitrogen), 100 units/ml penicillin, 100 µg/ml streptomycin and 2 mM L-glutamine.

**SECTION H: R-SCRIPT FOR REAL TIME PCR ANALYSIS (WITH MULTIPLE HOUSEKEEPING GENES NORMALIZATION AND MULTIPLE GROUP COMPARISONS)**

#Realtime PCR Analysis

#(for multiple housekeeping genes normalization and multiple groups comparisons)

#

#The user needs to read the lines where there are 3 hashes and alter as advised

#

### Set this directory to suit your data

>dataDir <- "C:\"

#

>setwd(dataDir)

#Just as a check, list the files in this directory

>list.files()

#load the lima package

>library(limma)

#

### Set these values to suit your data

>numTissues <- 4

>numRefGenes <- 1

>numReplicates <- 3

###Set the data filename

>dataFilename <- "data.txt"

# Assume the input files have row names in the first column...hence the row.names=1 parameter

# read in the ratios of control to reference, treatment to reference

>ratios <- read.table(file=dataFilename, row.names=1, header=TRUE, sep="\t")

#

#creat a weights matrix of right dimensions and set all weights to 1

>weights <- matrix(1, nrow=dim(ratios)[1], ncol=dim(ratios)[2])

# if any data values = NA, set weight for that data value to 0

>weights[is.na(ratios)] <- 0

#

#

>tissue <- factor(rep(1:numTissues, each=numRefGenes*numReplicates))

# If there were 4 Tissues, 1 RefGene and 3 Replicates, then the tissue factor would be

# [1] 1 1 1 1 2 2 2 2 3 3 3 3 4 4 4 4

# Levels: 1 2 3 4

#

>tissue

#

>referenceGene <- factor(rep(rep(1:numRefGenes, each=numReplicates), numTissues))

# If there were 1 refGenes and 3 Replicates and 4 Tissues, the factor referenceGene would be

# [1] 1 1 1 1 1 1 1 1 1 1 1 1 1 1 1 1

# Levels: 1 2 3

>referenceGene

#

# Make a design matrix. We want estimates of the mean ratio for control,

# and the mean ratio for treatment, so we won't fit an intercept term.

# We fit an effect for referenceGene, which is a nuisance parameter affecting

# our estimates of control and treatment, but which we don't really care about

#

>mm <- model.matrix(~ -1 + tissue)

#

### You need to enter abbreviations for each Tissue(Treatment), T1 to T4 in this example

>colnames(mm) <- c("T1", "T2","T3", "T4")

#

# fit the model to the logged ratios

#

>fit <- lmFit((ratios), design=mm,weights=weights)

#

# now fit the all effects

# make a contrast matrix

### You need to enter all possible contrasts betweeen tissues

>contrastsMatrix <- makeContrasts("T1-T2","T1-T3","T1-T4","T2-T3","T2-T4","T3-T4", levels=mm)

# fit the contrast

>fit2 <- contrasts.fit(fit, contr=contrastsMatrix)

>fit3 <- eBayes (fit2)

# calculate group-wise "means" and standard errors. Note: stderrs come

# from linear model, not from population

>estimates <- fit$coef[,1:numTissues]

>stdevs <- (fit$stdev*fit$sigma)[,1:numTissues]

>colnames(stdevs) <- paste(colnames(stdevs), "stdv")

#

#Create a toptable showing all contrasts. Use argument "genelist" of

#topTable to get estimates and stdvs into table (this is a hack).

>est <- data.frame(estimates, stdevs)

>ttallc <- topTable(fit3, adjust="BH", number=nrow(fit3$p.value), genelist=est)

#

#create a topTable showing details for each contrast

###You need to modify these lines to suit your tissue contrasts

>ttt1t2 <- topTable(fit3, adjust="BH", number=nrow(fit3$p.value),coef=1)

>ttt1t3 <- topTable(fit3, adjust="BH", number=nrow(fit3$p.value),coef=2)

>ttt1t4 <- topTable(fit3, adjust="BH", number=nrow(fit3$p.value),coef=3)

>ttt2t3 <- topTable(fit3, adjust="BH", number=nrow(fit3$p.value),coef=4)

>ttt2t4 <- topTable(fit3, adjust="BH", number=nrow(fit3$p.value),coef=5)

>ttt3t4 <- topTable(fit3, adjust="BH", number=nrow(fit3$p.value),coef=6)

#

# to write results to a file use something like

>write.table(file="t1t2.xls", ttt1t2, sep="\t", quote=F)

>write.table(file="t1t3.xls", ttt1t3, sep="\t", quote=F)

>write.table(file="t1t4.xls", ttt1t4, sep="\t", quote=F)

>write.table(file="t2t3.xls", ttt2t3, sep="\t", quote=F)

>write.table(file="t2t4.xls", ttt2t4, sep="\t", quote=F)

>write.table(file="t3t4.xls", ttt3t4, sep="\t", quote=F)

>write.table(file="means_std_allgroups.xls", est, sep="\t", quote=F)

#
